# Beyond Sabatier: Multiplicate Spillover Phenomena for Manipulating Catalytic Dynamics in Various Electrocatalysis

**DOI:** 10.1002/advs.202520177

**Published:** 2026-02-03

**Authors:** Di Wang, Ziyou Dong, Qianqian Yao, Shixue Dou, Jun Mei, Ziqi Sun

**Affiliations:** ^1^ Key Laboratory for Special Functional Materials of Ministry of Education National & Local Joint Engineering Research Center for High‐efficiency Display and Lighting Technology School of Nanoscience and Materials Engineering Henan University Zhengzhou 450046 China; ^2^ Institute of Energy Materials Science University of Shanghai for Science and Technology 516 Jungong Road Shanghai 200093 China; ^3^ Institute for Superconducting and Electronic Materials University of Wollongong Wollongong New South Wales 2522 Australia; ^4^ School of Chemistry and Physics Centre for Materials Science Queensland University of Technology 2 George Street Brisbane QLD 4000 Australia

**Keywords:** carbon, electrocatalysis, hydrogen, oxygen, spillover

## Abstract

Spillover phenomenon, which is characterized by the dynamic migration of active species across catalyst surfaces, provides a promising avenue to overcome the limitations dictated by the Sabatier principle in conventional catalysis. This comprehensive review highlights recent progress in multiplicate spillover, including hydrogen, oxygen, hydroxyl, and intermediate spillover, and their critical roles in modulating intermediate adsorption, strengthening interfacial transport, and manipulating catalytic dynamics. Then, various spillover‐mediated electrocatalytic reactions, such as hydrogen and oxygen evolution, carbon dioxide and nitrogen reduction, and methanol oxidization reactions, are systematically summarized. Finally, current challenges of different spillover systems are critically assessed and future research directions are briefly outlined. It is expected that this review offers some useful guidance on rational design of high‐performance spillover‐based electrocatalysts and promote advancement of sustainable energy conversion technologies.

## Graphical abstract

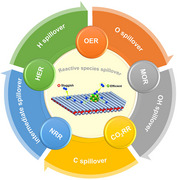

## 1 Introduction

Electrocatalysis has emerged as one of the cornerstones for clean energy conversion and storage, which acts as crucial roles in several energy‐related fields, such as hydrogen utilization and electrochemical transformations.^[^ ^1^ ^]^ For decades, the rational design of catalysts has been guided by a fundamental principle: the Sabatier principle, which indicates that the adsorption strength of reaction intermediates at active sites should remain moderate, and this means that excessively strong or weak binding inevitably leads to reduced catalytic activity.^[^ ^2^, ^3^ ^]^ This principle effectively creates a “volcano plot”, in which peak catalytic activity is achieved by a narrow range of materials with intermediate adsorption energies.^[^ ^4^ ^]^ This volcano‐type relationship renders it challenging for a single material to optimally facilitate all elementary steps of a complex reaction network, thereby limiting the advancement of electrocatalytic technologies.^[^ ^5^ ^]^

In the request to overcome these constraints and break the scaling relations dictated by Sabatier, the scientific community has turned their attention to dynamic interfacial phenomena.^[^ ^6^ ^]^ Spillover phenomena are primarily observed in hydrogenation reactions on metal‐supported catalysts.^[^ ^7^ ^]^ It was noticed that hydrogen gas could dissociate on a metal nanoparticle (e.g., Pt or Pd) and the resulting hydrogen atoms could subsequently spillover onto the adjacent oxide support (e.g., TiO_2_ or Al_2_O_3_), which could dramatically expand active surface area and modify local chemical environment.^[^ ^8^ ^]^ This discovery has initiated and blossomed into a rich field of research area, in which hydrogen is expanded to other species, such as oxygen and carbon monoxide (CO), and the recent various intermediates in electrocatalysis. Basically, the mechanistic process of spillover involves a sequence of key steps: i) the activation of a molecule (e.g., H_2_, O_2_, H_2_O, or CO_2_) and its dissociation into active species on an active phase (often a metal or a metal compound); ii) the migration of these mobilized species across the interface between the active phase and the receptor phase, which could be a carbon material, metal oxide, or another metal; and iii) the transport and subsequent participation of these species in surface diffusion, reaction, or adsorption/desorption modulation on the receptor surface. Intrinsically, this whole process is governed by the kinetics of surface diffusion and the strength of the interaction between the spilled species and the receptor surface. Through the migration of active species from a primary site onto a secondary surface, the spillover process could decouple the activation site from the reaction site, which effectively produce a synergistic system in which different components of a catalyst system perform specialized tasks.

On the other hand, the implications of spillover phenomenon for electrocatalysis are profound.^[^ ^9^ ^]^ Most electrocatalytic reactions are relatively complex, in which multi‐step proton‐coupled electron transfers and various adsorbed intermediates (e.g., *H, OOH, or COOH) are involved.^[^ ^10^ ^]^ Considering that the binding strength of these intermediates is the primary determinant of catalytic activity, spillover effects offer a powerful pathway to manipulate adsorption energies toward target goals. Furthermore, spillover can accelerate interfacial mass transport for increasing catalyst utilization and mitigating diffusion limitations in heterogeneous reactions, particularly in electrochemical systems in which reactant concentration at the electrode‐electrolyte interface possess considerable influences on overall reaction dynamics. Hence, spillover effects have received intensive attention in electrocatalysis with major progress has been achieved for different spillover systems with different catalysts and reaction pathways.

**Figure 1:**
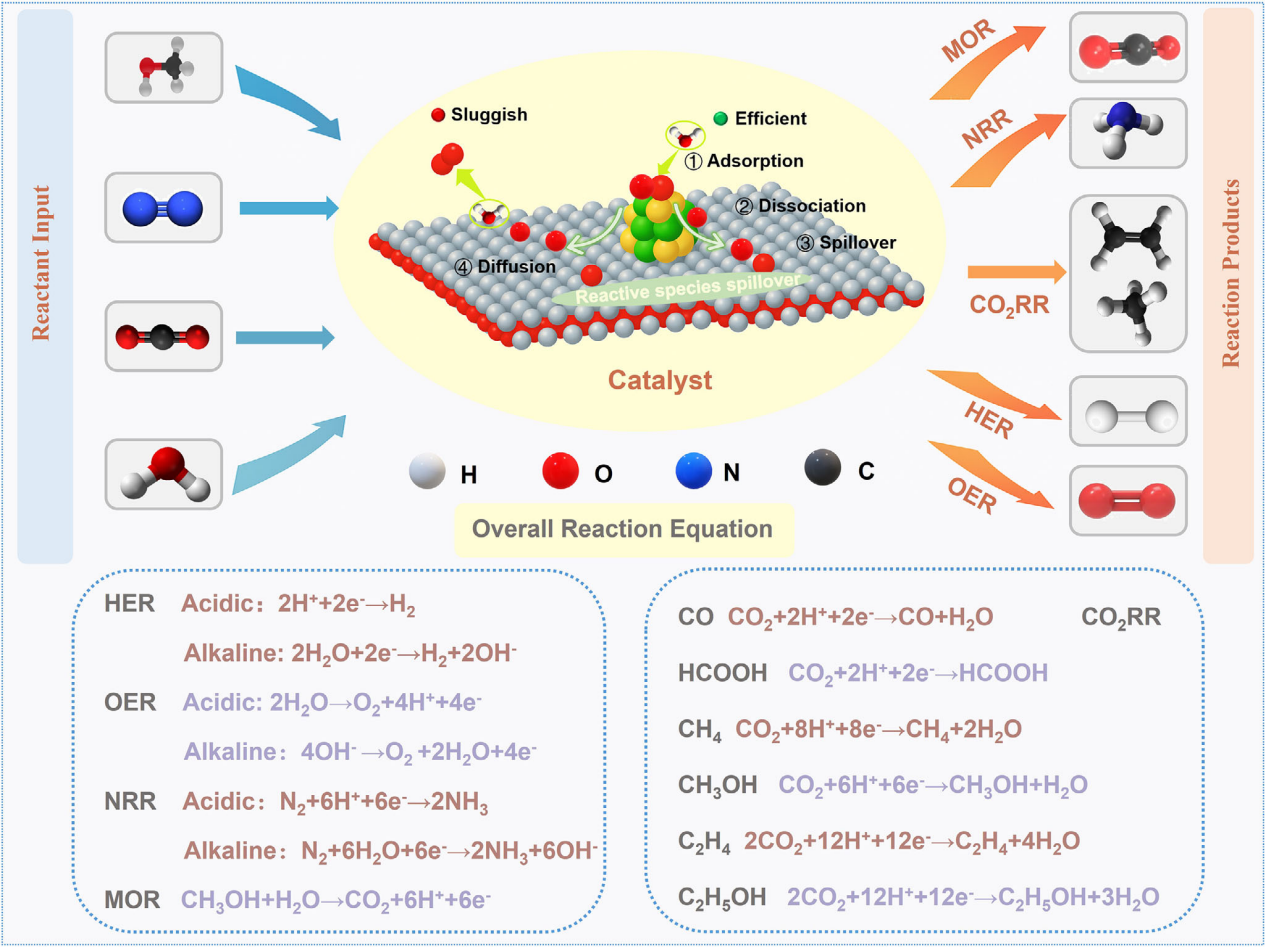
Schematic illustration of spillover phenomena for electrocatalytic reactions.

Unlike previous reviews that primarily emphasized hydrogen or oxygen spillover,^[^ ^11^, ^12^, ^13^, ^14^ ^]^ in this review, as illustrated in **Figure** **1**, a comprehensive overview is provided on recent progress in multiplicate spillover, including hydrogen, oxygen, hydroxyl, and intermediate spillover, and their critical roles in modulating adsorption, strengthening interfacial transport, and manipulating catalytic dynamics, are highlights. Then, various spillover systems based on precious metal and transition metal‐based catalysts are discussed, which is followed by a detailed review on diverse spillover‐mediated electrocatalytic reactions, such as hydrogen evolution reaction (HER), oxygen evolution reaction (OER), carbon dioxide reduction reaction (CO_2_RR), nitrogen reduction reaction (NRR), and methanol oxidization reaction (MOR). Finally, the strengths and limitations of current spillover systems are critically assessed and future research directions are briefly outlined. It is expected that this timely review offers some theoretical and experimental guidance on rational design of highly efficient electrocatalysts based on spillover and promote advancement of sustainable energy conversion technologies.

## 2 Historical Outline of Spillover

**Figure 2:**
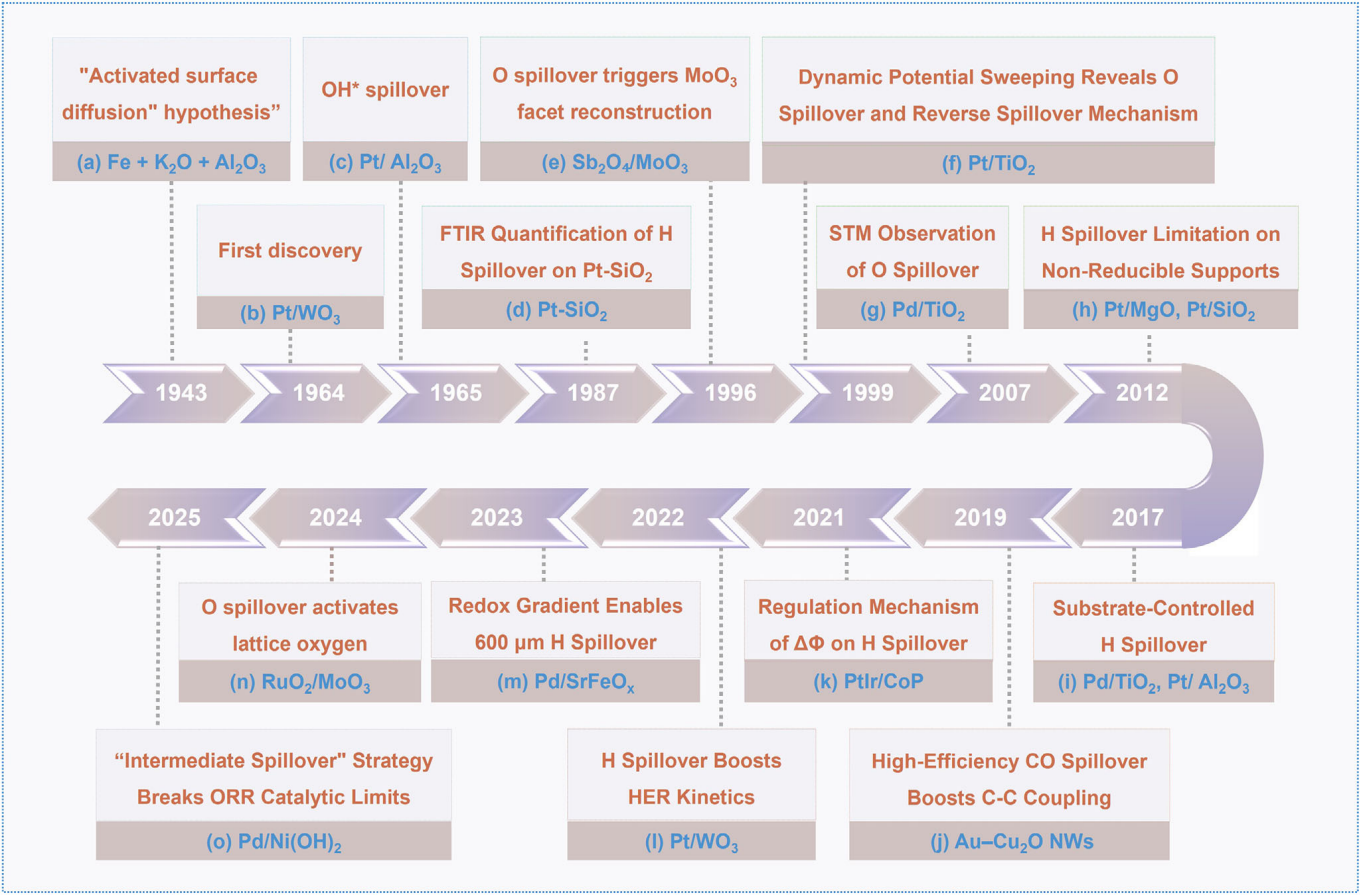
Timeline‐based research progress associated with spillover in catalysis.

As illustrated in **Figure** **2**, the conceptual foundation of spillover can be traced back to the 1943s, as indicated by the activated surface diffusion theory, in which reactants could migrate across catalyst surface when catalyst particles are smaller than diffusion length, surpassing conventional gas‐phase limitations.^[^ ^15^ ^]^ In 1964, it was experimentally observed that H_2_ molecules dissociated on Pt and the resulting atomic hydrogen migrated to WO_3_ in the Pt/WO_3_ system, inducing the characteristic coloration of tungsten bronzes.^[^ ^16^ ^]^ This pioneering work confirms the interfacial migration of hydrogen species and also broadens the research scope of metal‐support interactions. Carter et al. provided the first isotopic evidence of hydroxyl spillover.^[^ ^17^ ^]^ Subsequently, in 1969, hydrogen spillover was formally defined as the physicochemical process in which hydrogen atoms that were generated via dissociative adsorption on noble metals migrated onto the adjacent oxide supports.^[^ ^18^ ^]^ Candau and Conner quantified hydrogen spillover kinetics on Pt‐SiO_2_ with the assistance of fourier‐transform infrared spectroscopy (FT‐IR), demonstrating the spillover step as the rate‐determining process.^[^ ^19^ ^]^

Since the 1990s, spillover‐related research has achieved systematic breakthroughs driven by the development of nanosized catalysts and the innovation of advanced characterization techniques. The scope of spillover systems has expanded from classical metal‐oxide binary systems to complex heterointerfaces such as metal‐carbon and oxide‐oxide composites, while the types of spillover species have extended beyond hydrogen to include O*, OH*, and CO* intermediates. For instance, Gaigneaux et al. revealed that oxygen spillover from α‐Sb_2_O_4_ could induce the surface reconstruction of MoO_3_, unveiling a cooperative mechanism in which interfacial oxygen migration modulated active sites.^[^ ^20^ ^]^ Lin et al. further demonstrated oxygen spillover and reverse spillover in Pt/TiO_2_ catalysts, and found that the increased Pt loading enhanced the process and hydrogen pretreatment transformed the rate‐limiting step from oxygen diffusion to electrochemical adsorption/desorption.^[^ ^21^ ^]^ Sen et al. confirmed that interfacial H‐CO spillover complexes in Ni/Al_2_O_3_ play a crucial role in methane formation.^[^ ^22^ ^]^ Bowker et al. employed scanning tunneling microscopy (STM) technique to visualize oxygen spillover and confirmed the origin from the differences in dissociation capabilities of Pd/TiO_2_.^[^ ^23^ ^]^ Later, Kyriakou et al. (2012) evidenced that isolated Pd atoms could efficiently dissociate H_2_, while a Cu substrate could provide weak hydrogen binding, enabling synergistic spillover for achieving selective hydrogenation of styrene and acetylene.^[^ ^24^ ^]^ Prins et al. found that hydrogen atoms could not migrate onto defect‐free surfaces of non‐reducible supports (e.g., MgO or SiO_2_), but feasibly migrate via surface defects, carbon deposits, or reducible oxides (e.g., WO_3_).^[^ ^25^ ^]^ Karim et al. verified that reducible supports (e.g., TiO_2_) enabled efficient long‐range hydrogen spillover via coupled proton‐electron transfer, while non‐reducible supports (e.g., Al_2_O_3_) were restricted by the metal‐water interaction.^[^ ^26^ ^]^ In 2019, Grätzel et al. realized ≈95% CO spillover efficiency at Ag‐Cu nanowire interfaces, which promoted selective C─C coupling.^[^ ^27^ ^]^ Li et al. revealed that smaller work function differences (Δϕ) across interfaces could significantly lower proton adsorption barriers and promote hydrogen transfer.^[^ ^28^ ^]^ Kamada et al. reported hydrogen spillover over distances in micrometer scale (up to 600 µm) in Pd/SrFeO_x_ systems, and concluded that the driving force was the released energy (200 kJ mol^−1^) associated with the reduction of Fe^4+^, while the redox gradient between Fe^4+^/Fe^3+^ couples promoted the continuous migration, revealing that redox‐driven transport underpins long‐range spillover.^[^ ^29^ ^]^ Bai et al. demonstrated the hydrogen spillover distance beyond 50 nm in Zn‐ZIF‐8 through water‐assisted and ligand‐functionalized strategies.^[^ ^30^ ^]^ Most recently, Wang et al. found the intermediate spillover in Pd/Ni(OH)_2_ interfaces, which could decouple O_2_ activation from OH* reduction, achieving a half‐wave potential increase of 70 mV in oxygen reduction reaction (ORR).^[^ ^31^ ^]^

These studies establish a comprehensive theoretical and experimental framework for spillover phenomena, which is beneficial to composite catalyst design in electrocatalysis. Importantly, hydrogen spillover is not viewed as a simple migration process but rather as a dynamic phenomenon that could reshape intermediate adsorption behaviors and reconstruct interfacial microenvironments, enabling accurate control of catalytic pathways and kinetics. Mechanistically, spillover enhances electrocatalysis through three primary effects: i) reconstruction of electronic structures at active sites to optimize intermediate adsorption; ii) facilitation of interfacial mass transport for accelerating reaction kinetics; and iii) stabilization of dynamic catalytic interfaces. In electrocatalysis, spillover offers the potential to transcend the limitations imposed by adsorption energetics, allowing the formation of dynamic active centers that cooperatively motivate multistep complex catalytic reactions.

## 3 Classification Categories of Spillover

### 3.1 Fundamental Principles

A comprehensive understanding of the spillover phenomenon in heterogeneous catalysis requires moving beyond traditional geometry‐based models to uncover its intrinsic electronic nature and energy‐driven mechanisms. Two fundamental electronic parameters, including bandgap and work function difference, are often used to define the physical basis of spillover behavior. Essentially, the migration of active species from donor to acceptor phases originates from electronic synergism between the constituent materials. An in‐depth understanding and precise regulation of these two parameters are prerequisites for the rational design of high‐performance catalysts.

The work function difference at the metal‐support interface provides the primary driving force for spillover. When two materials come into contact, electrons spontaneously transfer from the phase with a lower work function to that with a higher one until Fermi level equilibration is achieved.^[^ ^28^ ^]^ This charge redistribution induces the formation of a localized electric field and a space charge region at the interface. In these systems composed of a high work function metal and a low work function semiconductor, a slight accumulation of negative charge occurs on the metal side, while the support becomes positively polarized. This interfacial charge configuration exerts three primary effects on the spillover process: i) the resulting electrostatic potential gradient significantly lowers the activation energy for charged species crossing the phase boundary; ii) the oriented electric field provides a well‐defined migration pathway; and iii) interfacial charge transfer modifies the electronic states of surface atoms on the support, thus enhancing their reactivity as acceptor sites.

Beyond the initial electronic driving force, the band structure characteristics of the acceptor support determine its capacity to accommodate migrated species. Engineering the bandgap through doping, defect, or size modulation serves as a critical strategy for optimizing support functionality.^[^ ^32^, ^33^ ^]^ Specifically, the introduction of defect states within the bandgap not only provides thermodynamically stable trapping centers for spillover intermediates but also facilitates electron exchange between the support and the adsorbed species. Moreover, these localized states act as efficient electronic channels that promote surface diffusion of migrated species to extend the spatial coverage of active regions.

Generally, the formation of a spillover process fundamentally relies on the coexistence of appropriate spillover species, a well‐matched donor/acceptor pair, and a conductive heterointerface that bridges them. Typically, the donor refers to metallic active sites (e.g., Pt, Ni, Cu, or Co), which possess strong adsorption and activation capabilities toward small molecules, thus generating mobile intermediates that are commonly referred as spillover species, such as H*, O*, CO*, or OH*. The acceptor usually consists of a metal (hydro)oxide (e.g., TiO_2_, CeO_2_, or WO_3_) or other support material, which provides adjacent low‐energy sites capable of capturing and stabilizing these intermediates. In the meanwhile, the heterointerface between the donor and acceptor plays a crucial role, which enables electronic coupling and establishes a local potential gradient that drives the directional migration of the spillover species. Therefore, the efficiency of spillover is governed by the adsorption energy matching between the donor and the acceptor, the continuity of migration pathways (e.g., via vacancies or conductive bridges), and the strength of interfacial charge‐transfer capability that facilitates charge redistribution during the transfer.

### 3.2 Hydrogen Spillover

Hydrogen spillover essentially refers to the dynamic migration of reactive hydrogen species across heterogeneous interfaces, which proceeds through four fundamental steps: i) molecular hydrogen undergoes chemisorption and dissociation into atomic hydrogen at metallic active sites; ii) dissociated hydrogen atoms diffuse across metal surface toward metal‐support interface; iii) hydrogen atoms overcome interfacial energy barrier to transfer from metallic phase to the support; and vi) migrated species further diffuse along support surface to reach a dynamic equilibrium.^[^ ^34^ ^]^ This multistep process involves complex interfacial electronic interactions, in which the transport efficiency is governed by metal‐support bonding strength, interfacial defect states, and surface properties of the support. Critically, the Fermi‐level mismatch of metal and support introduces an electronic migration barrier at the interface, which constitutes the primary limitation to hydrogen spillover kinetics.^[^ ^35^, ^36^, ^37^ ^]^

**Figure 3:**
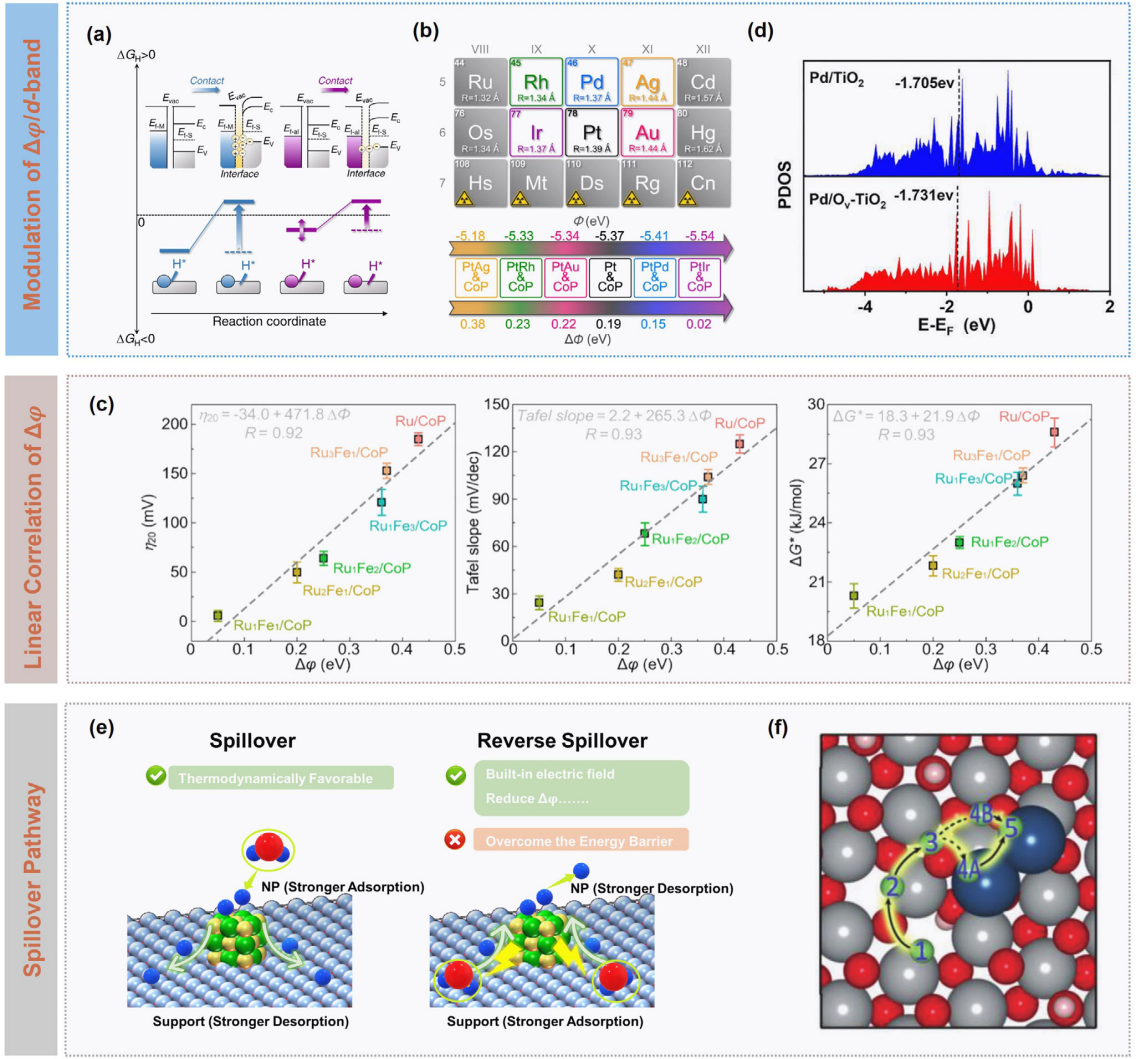
Catalytic dynamics modulation on hydrogen spillover. a) Schematic illustration of interfacial electronic configurations and hydrogen spillover phenomenon in binary catalysts. b) Design of PtM/CoP model catalysts with the controllable ΔΦ. Reproduced under a Creative Commons Attribution License 4.0 International License.^[^ ^28^ ^]^ Copyright 2021, The Authors. c) Plots of η_20_, dependence of Tafel slope and ΔG* on ΔΦ. Reproduced with permission.^[^ ^38^ ^]^ Copyright 2022, American Chemical Society. d) Projected density of states (PDOS) plots for Pd/O*_v_*‐TiO_2_ and Pd/TiO_2_. Reproduced with permission.^[^ ^39^ ^]^ Copyright 2023, American Chemical Society. e) Schematic illustration of spillover and reverse spillover. f) Schematic illustration of hydrogen spillover pathways for Pt atom dimers. Reproduced with permission.^[^ ^41^ ^]^ Copyright 2023, Wiley.

To overcome the thermodynamical bottleneck, Li et al. proposed binary cooperative catalysts that integrated metal components with strong hydrogen adsorption capability (∆G_H*_ < 0) and support components with favorable hydrogen desorption characteristics (∆G_H*_ ≥ 0), thus enabling complementary functions beyond the constraints of single‐component catalysts. Systematic studies revealed that the work function difference (**Δ**∅) between metal and support was the determining parameter governing charge redistribution and spillover energetics.^[^ ^28^ ^]^ As shown in **Figure** **3**a, a large ∆∅ induces Schottky junction formation with accumulated interfacial charge, enhancing proton affinity but simultaneously elevating spillover barrier; in contrast, while a smaller ∆∅ reduces interfacial charge polarization, thereby lowering spillover kinetic barrier. Guided by the principle in Figure 3b, alloying strategies were developed to precisely tune work function of noble metals (e.g., Ir, Rh, Pd, Ag, and Au) alloyed with Pt, yielding optimal matching with CoP supports. It was concluded that the PtIr/CoP system exhibited significantly reduced spillover barriers and improved interfacial charge transfer efficiency, achieving superior spillover dynamics.^[^ ^28^ ^]^ Furthermore, quantitative correlations were established between ∆∅ and catalytic parameters (η_20_, Tafel slope, ***Δ*** G*), confirming the critical role of ∆∅ in regulating hydrogen spillover kinetics (Figure 3c).^[^ ^38^ ^]^ Other strategies, such as oxygen‐vacancy engineering in Pd/O*_v_*‐TiO_2_ (Figure 3d), demonstrated that defect‐induced electronic modulation on *d*‐band center promoted efficient hydrogen spillover while mitigating catalyst poisoning.^[^ ^39^ ^]^ Yang et al. engineered a Ni/MoO_x_H_y_ heterostructure via hydrogen doping and oxygen vacancies for reducing ∆∅ and promoting H migration, while maintaining Ni in its metallic state.^[^ ^40^ ^]^

As compared in Figure 3e, in conventional spillover pathways, metal sites should mediate hydrogen adsorption and desorption simultaneously, thus inevitably compromising one aspect. In contrast, the reverse spillover mechanism can achieve functional separation and synergistic enhancement. Specifically, the support serves as the primary site for water activation and O─H bond cleavage, which is a critical step for overcoming sluggish water dissociation in alkaline HER, while metallic active sites with near‐thermoneutral ∆G_H*_ facilitate hydrogen recombination and desorption. For instance, Lin et al. demonstrated that Pt atom dimers anchored on NiOOH supports could effectively mediate reverse spillover, as illustrated in Figure 3f, in which strong Pt─O interactions could stabilize atomic dispersion, enabling electronic regulation at the interface, and hydrogen species dissociated on NiOOH would undergo a low‐barrier reverse transfer to Pt dimers.^[^ ^41^ ^]^ Guo et al. developed coral‐like Pd/CoNiP heterostructures via electrodeposition‐oxidative etching, in which interfacial electronic reconstruction optimized water activation at CoNiP while tuning Pd‐H binding, and found that the resulting reverse spillover accelerated alkaline HER kinetics.^[^ ^42^ ^]^

Despite these promising advances, reverse spillover remains a complex process governed by the interplay of interfacial structure, electric field, and intrinsic properties of electrocatalysts. Direct visualization of intermediate migration under operating electric fields remains highly challenging, and the phenomenon has been demonstrated primarily in model‐based systems. Furthermore, the strong adsorption of intermediates under high polarization risks support saturation or poisoning, while long‐term stability of both support and metal components remains an unresolved issue. Nevertheless, the emergence of reverse spillover provides a powerful design paradigm for catalyst engineering, which can highlight the importance of spatially and functionally separated active sites in electrocatalysis.

### 3.3 Oxygen Spillover

Oxygen spillover describes the directional migration of reactive oxygen species (ROS, e.g. *O or *OOH) between metallic active sites and supports. Unlike conventional gas‐phase transport, this process relies on surface‐mediated diffusion, enabling nominally inert supports to stabilize and shuttle oxygen species. Such dynamic redistribution prevents active‐site poisoning by excessive adsorption while enhancing utilization efficiency of oxygen intermediates, thus promoting both catalytic activity and durability.^[^ ^11^, ^43^ ^]^ In OER, oxygen spillover facilitates the transfer of O‐based intermediates from metals to supports, alleviating site blockage, stabilizing ROS, lowering overpotentials, and suppressing metal over‐oxidation and dissolution.^[^ ^44^ ^]^ In ORR, the transferred species help mitigate peroxide accumulation and Fenton side reactions, improving selectivity for the four‐electron pathway while enhancing catalyst durability.^[^ ^45^ ^]^

**Figure 4:**
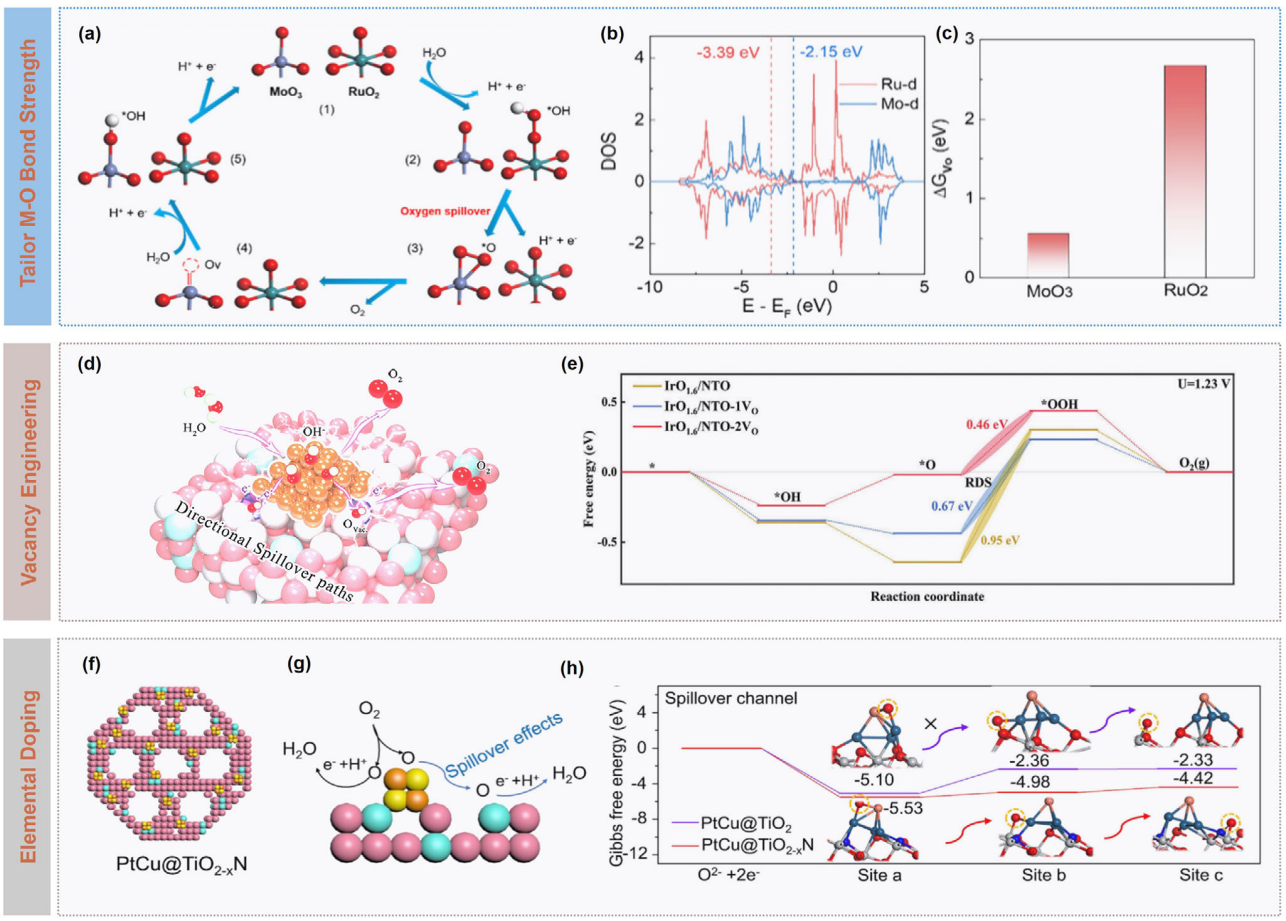
Catalytic dynamics modulation on oxygen spillover. a) Schematic illustration of the proposed oxygen spillover in the RuO_2_/MoO_3_ catalysts for OER in acid. b) Theoretical density of states (DOS) plots and c) calculated energy for the formation of oxygen vacancy in RuO_2_ or MoO_3_ of the RuO_2_/MoO_3_ heterojunction. Reproduced with permission.^[^ ^47^ ^]^ Copyright 2024, Royal Society of Chemistry. d) Schematic illustration of the spillover pathway formed in the Ir/Nb‐doped TiO_2_ catalyst, and e) Gibbs free energy plots. Reproduced with permission.^[^ ^49^ ^]^ Copyright 2025, Wiley. f) Schematic of catalytic structure and g) spillover mechanism of PtCu@TiO_2_‐*_x_* N catalyst, and h) Free energy diagram for spillover process on PtCu@TiO_2_‐*_x_* N and PtCu@TiO_2_. Reproduced with permission.^[^ ^50^ ^]^ Copyright 2024, Elsevier.

Mechanistically, the efficiency of oxygen spillover depends strongly on interfacial electronic coupling. Optimal Fermi‐level matching between metal and support forms efficient charge‐transfer channels, while the mediated M─O bond strength ensures both feasible spillover and subsequent catalytic turnover.^[^ ^46^ ^]^ For example, Guo et al. developed a RuO_2_/MoO_3_ heterojunction catalyst exemplifying an effective oxygen spillover strategy (**Figure** **4**a).^[^ ^47^ ^]^ By leveraging the high *d*‐band center (Figure 4b) and the low oxygen‐vacancy formation energy of MoO_3_ (Figure 4c), active oxygen species were directed from RuO_2_ toward MoO_3_. It is concluded that efficient oxygen spillover requires two main conditions: i) the M─O bond strength in the acceptor phase (e.g., MoO_3_) exceed the Ru─O* bond to ensure thermodynamic feasibility; ii) the oxygen‐vacancy formation energy of the acceptor oxide should be lower than that of RuO_2_, enabling lattice oxygen to actively participate in the OER.

The structure and chemical composition of catalytic materials play crucial roles in modulating oxygen spillover efficiency. Controlled introduction of oxygen vacancies has been demonstrated to optimize the dynamic behavior of catalysts.^[^ ^48^ ^]^ These vacancies not only provide low‐energy migration pathways for oxygen species but also adjust local coordination environments and electronic states of metal sites. Moreover, oxygen vacancies serve as effective electron‐transport mediators, significantly facilitating interfacial charge transfer. For instance, in the Ir/TiO_2_ system, Zhu et al. showed that vacancy‐induced electronic channels promote OH^−^ migration from Ir sites to the TiO_2_ support (Figure 4d), reducing the energy barrier of the rate‐determining OER step from 0.95 to 0.46 eV and enhancing reaction kinetics and spillover efficiency (Figure 4e).^[^ ^49^ ^]^ This defect‐engineering strategy highlights the importance of precisely controlling vacancy type, concentration, and distribution to rationally direct oxygen migration pathways. High‐surface‐area mesoporous structures further facilitate bulk diffusion of oxygen species, while dopants such as Cr, Fe, or N can introduce Lewis acidic sites or induce lattice distortions, thereby fine‐tuning the electronic band structure and reaction energy barriers. For example, Wang et al. developed a 3D ordered mesoporous nitrogen‐doped TiO_2_ (TiO_2_‐*_x_* N) support (Figure 4f), in which hierarchical porosity and electronic modulation produced a highly conductive and oxygen‐vacancy‐rich platform for spillover effects (Figure 4g). As illustrated in Figure 4h, this design reduced the interfacial charge transfer barrier from 2.74 to 0.56 eV and induced electron‐deficient Pt sites, lowering adsorption energies by ≈30%.^[^ ^50^ ^]^ Besides, interfacial chemical environment exerts significant influence on oxygen spillover. Systematic studies demonstrated that alkaline conditions could favor OH^−^‐mediated proton transfer and stabilize high‐valent metal intermediates, thus facilitating Co^3+^ to Co^4+^ conversion and enhancing reaction kinetics, whereas acidic environments with high proton concentrations inhibit metal oxidation, thereby increasing reaction barriers.^[^ ^51^ ^]^

In spite of significant progress on oxygen spillover, some critical challenges remain. Direct visualization of oxygen migration is difficult, and rational construction of stable metal‐support interfaces is demanding. Moreover, mismatches between spillover rates and subsequent catalytic steps, intermediate poisoning, and long‐term structural stability limit the practical applications. Additionally, current mechanistic insights are largely based on theoretical systems, which should be reconciled with the complexity and scalability of industrial applications. To address these challenges, advanced characterization techniques, cross‐disciplinary cooperation, and innovative synthesis strategies are highly required to fully exploit oxygen spillover in electrocatalysis.

### 3.4 Hydroxyl Spillover

Hydroxyl (OH^−^) spillover refers to the directional migration of hydroxyl species from metal or metal‐oxide active sites toward supports or adjacent reactive sites, which fundamentally involves complex interfacial dynamics with coupled proton and electron transfer. This phenomenon is of much significance in various electrocatalytic reactions by modulating local reaction environment.^[^ ^52^ ^]^ In the OER, hydroxyl spillover allows OH^−^ to bypass the rate‐limiting *O to *OOH step, reducing overpotentials and enhancing electrode stability.^[^ ^53^ ^]^ In the ORR, spillover facilitates the removal of reactive oxygen species, mitigating catalyst degradation via Fenton‐type reactions.^[^ ^54^ ^]^ In alkaline HER, hydroxyl migration decouples *OH generation and consumption in space or time, alleviating pH‐induced kinetic limitations.^[^ ^55^ ^]^ Similarly, in CO_2_ reduction reactions (CO_2_RR), hydroxyl spillover removes adsorbed oxygenated species, preserving the reduced state of active metals and suppressing the competitive hydrogen evolution reaction, thereby improving selectivity and yield of carbon‐based products.^[^ ^56^ ^]^

**Figure 5:**
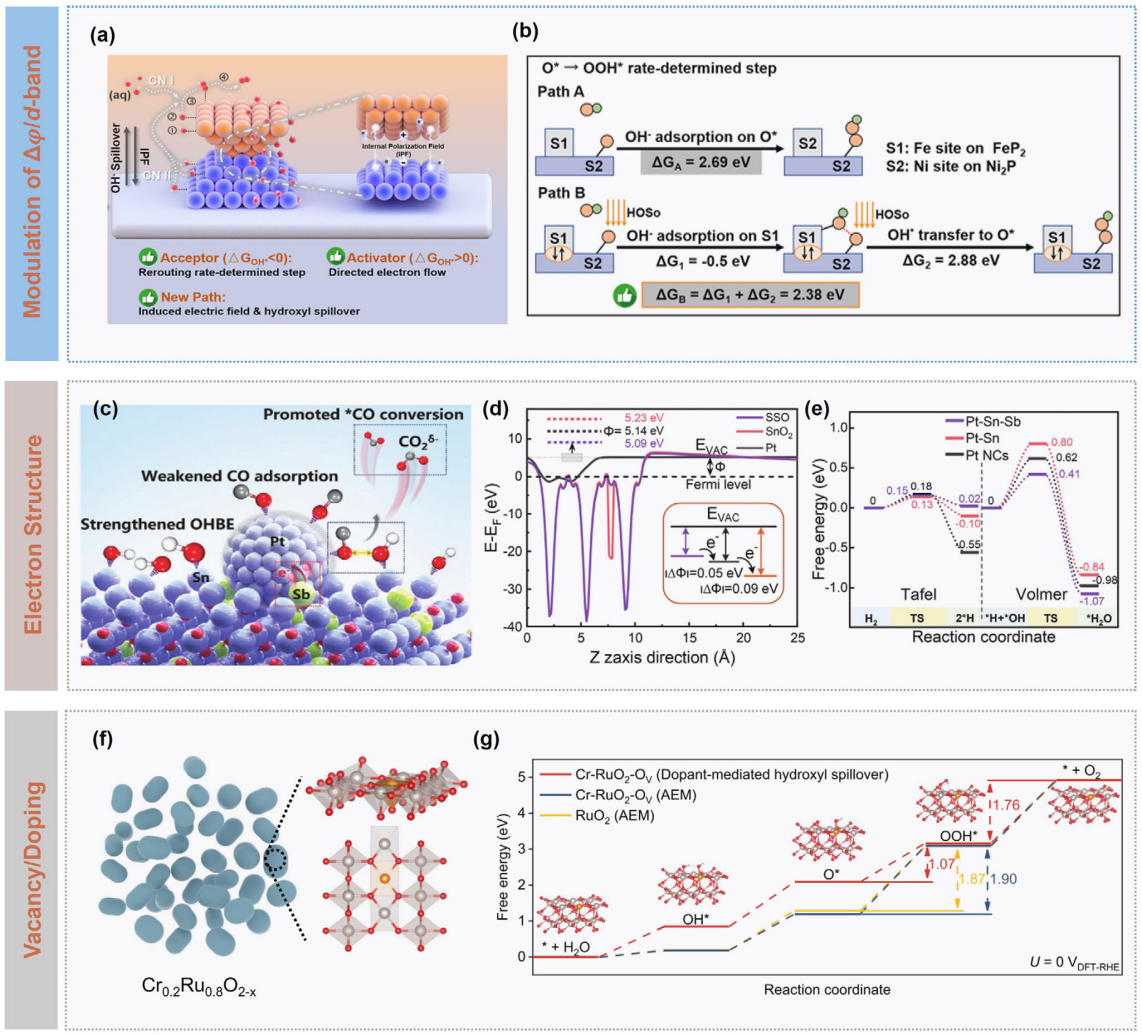
Catalytic dynamics modulation on hydroxyl spillover. a) Schematic illustration of catalyst construction for hydroxyl spillover in water electrolysis, and b) Gibbs free energy of the RDS of Ni_2_P/FeP_2_ along different reaction paths. Reproduced under a Creative Commons Attribution License 4.0 International License.^[^ ^57^ ^]^ Copyright 2023, The Authors. c) Schematic illustration of enhanced CO resistance mechanism on Pt‐Sn‐Sb, d) work functions of Sn_0.88_Sb_0.12_O_2_ (SSO), SnO_2_, and Pt, and e) Calculated energy profile for HOR on Pt–Sn–Sb, Pt–Sn, and Pt NCs. Reproduced with permission.^[^ ^58^ ^]^ Copyright 2024, Wiley. f) Schematic illustration of synthesis and structure of Cr_0.2_Ru_0.8_O_2‐x_ catalyst, and g) free energy profiles of RuO_2_ and Cr‐RuO_2_‐O*_v_* along different OER pathways. Reproduced under a Creative Commons Attribution License 4.0 International License.^[^ ^59^ ^]^ Copyright 2024, The Authors.

It is evidenced that the driving forces for hydroxyl spillover arise from a multiscale synergy between the built‐in interfacial electric fields, adsorption energy gradients, and electronic structure modulation. In heterostructured catalysts, the differences in work function between components generate significant internal polarization fields, thus creating an electrostatic potential gradient that drives hydroxyl migration. Concurrently, the differences in adsorption energy between donor and acceptor sites establish a thermodynamic driving force for OH^−^ migration. For instance, Xie et al. demonstrated that precise tuning of the Ni_2_P/FeP_2_ heterointerface (∆∅ = 0.158 eV) induced a strong internal polarization field for spontaneous OH^−^ adsorption at FeP_2_ (∆G_OH‐_ < 0) and efficient activation at Ni_2_P (∆G_OH‐_ > 0). As depicted in **Figure** **5**a, the dual‐channel hydroxyl supply mechanism comprises direct electrolyte provision and polarization‐driven migration, which can ensure robust OH^−^ delivery under varying electrolyte concentrations. Theoretical calculations indicate that the polarization field reduces hydroxyl migration barriers by 11.5 % and shifts the Ni *d*‐band center down by 0.24 eV, lowering the rate‐determining step (RDS) barrier from 2.69 to 2.38 eV for OER (Figure 5b).^[^ ^57^ ^]^ Wang et al. highlighted the role of electronic structure modulation in ultra‐small Pt cluster/Sb‐Sn oxide catalysts, in which interface‐driven electron back‐transfer reduced the Pt *d*‐band center, weakening *H and CO adsorption while enhancing OH^−^ adsorption (Figure 5c), and Sb acted as an electron buffer for Pt (Figure 5d), whereas Sn selectively adsorbed OH^−^, collectively lowering the hydroxyl spillover barrier to 1.07 eV and reducing activation energy of the Volmer step to 0.41 eV (Figure 5e).^[^ ^58^ ^]^

Defect engineering, such as introducing oxygen vacancies, can also modulate electronic effects that shift *d*‐band center and lower hydroxyl migration barriers. For example, it was reported that high hydroxyl mobility could be achieved through Cr‐Ru synergy in Cr‐doped RuO_2_ catalysts (Cr_0_._2_Ru_0.8_O_2‐x_, Figure 5f), in which Cr doping combined with oxygen vacancies shifted Ru *d*‐band center down by 0.35 eV, thus reducing hydroxyl spillover barriers to 1.07 eV (Figure 5g), and redirects the rate‐limiting step from the *OOH formation to a more favourable O_2_ evolution pathway, thereby overcoming high‐energy barriers (>1.8 eV) and Ru overoxidation under acidic conditions.^[^ ^59^ ^]^

These above‐mentioned multi‐synergistic strategies not only reduce migration barriers but also optimize intermediate adsorption, simultaneously enhancing catalytic activity and stability. Nevertheless, hydroxyl spillover faces significant challenges. Beyond these limitations in in situ characterization, catalyst design should balance interfacial construction with stable hydroxyl transport on supports. Ideal high‐efficiency interfaces require electronic structure matching and impose constraints on hydroxyl affinity, defect concentration, and crystallographic stability. Furthermore, active sites may gradually deactivate due to strong adsorption of intermediates, and the kinetic matching between hydroxyl migration and subsequent surface reactions directly affects the sustained catalytic efficiency and stability.

### 3.5 Carbon Spillover

Carbon spillover, which involves the dynamic migration of active carbon species (e.g., *CO) from metal active sites to supports or adjacent sites, is a typical process governed synergistically by interfacial electronic effects, support characteristics, and reaction conditions. Serving as a critical interfacial regulation mechanism, carbon spillover uniquely enhances product selectivity in catalytic reactions. By extending active sites from metals to support surface, carbon spillover not only increases utilization ratios of active sites but also reduces surface coverage ratios of intermediates on metals, thus buffering side reactions and steering the reaction pathway toward desired products. For instance, in CO_2_RR, carbon spillover stabilizes key intermediates such as *CO, prolonging their lifetimes and facilitating interfacial coupling to form multi‐carbon products, thereby enhancing C_2+_ selectivity.^[^ ^60^ ^]^ Similarly, in MOR and selective alcohol oxidation, carbon spillover modulates interfacial electronic structure and local chemical environment, suppressing complete oxidation and promoting partial oxidation pathways to favour high‐value products.^[^ ^61^, ^62^ ^]^ Moreover, spillover can expand the cooperative effects of supports, enabling reducible oxides or porous materials to participate in electron and species transport, thereby lowering reaction barriers and optimizing kinetics. Nevertheless, the practical implementation of carbon spillover remains challenging, which requires precise control over metal‐support interactions, defect states, and external‐field modulation.^[^ ^63^ ^]^

**Figure 6:**
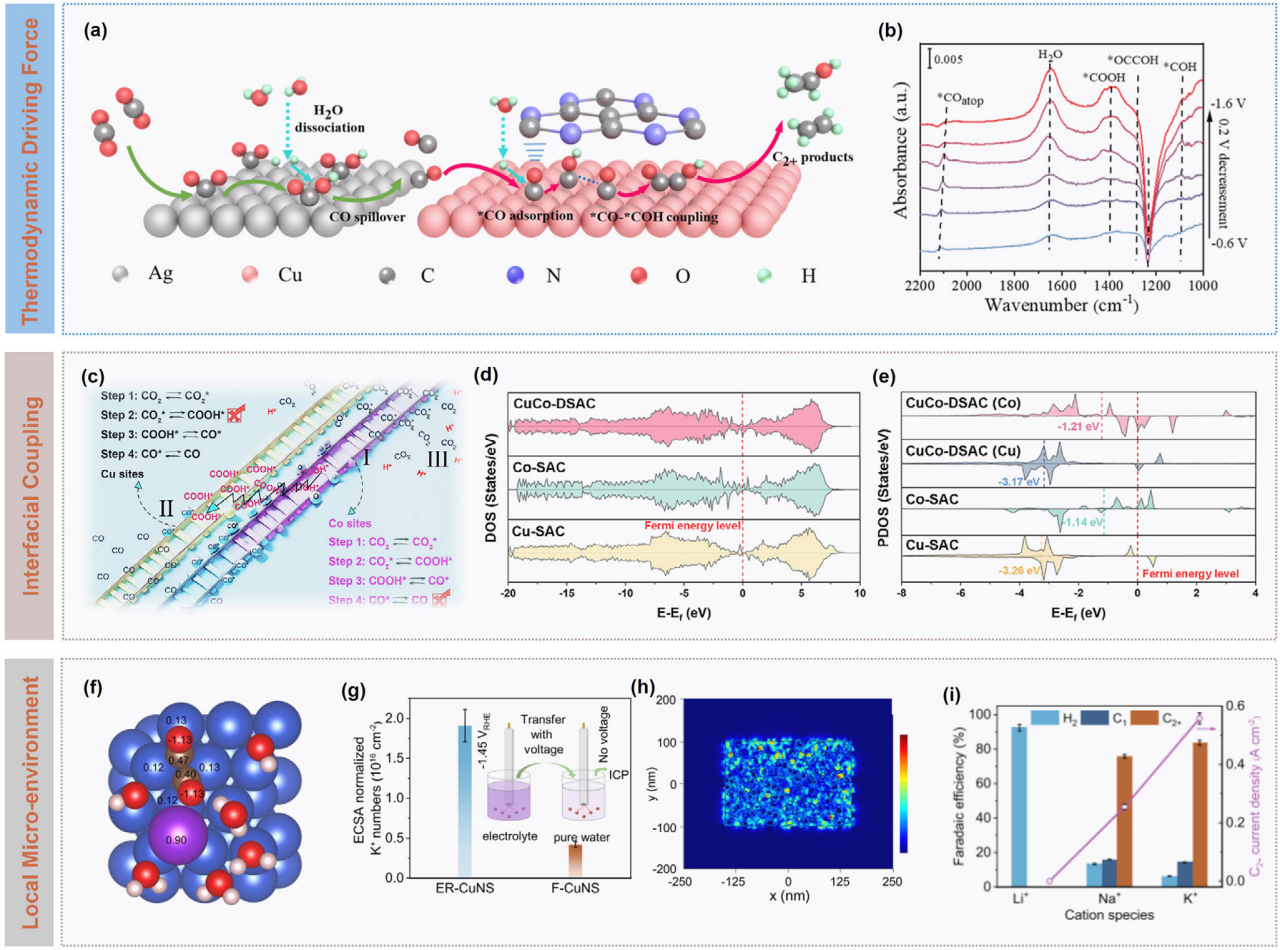
Catalytic dynamics modulation on carbon spillover. a) Schematic illustration of the Ag‐Cu dual‐site synergy: Ag sites for CO_2_‐to‐CO conversion and CO release, while adjacent Cu sites capture CO for further reduction, and b) in situ ATR‐SEIRAS spectra. Reproduced with permission.^[^ ^64^ ^]^ Copyright 2025, American Chemical Society. c) Schematic illustration of CO_2_ reduction pathways for copper and cobalt sites, Co sites activate CO_2_ and stabilize *COOH, while adjacent Cu sites facilitate CO desorption, enabling efficient spillover, and d) DOS and e) partial DOS (PDOS). Reproduced with permission.^[^ ^65^ ^]^ Copyright 2025, Wiley. f) Charge density analysis of *OCCO on Cu‐H_2_O‐K^+^ slab, and g) ECSA‐normalized K^+^ number and h) K^+^ distribution on ER‐CuNS catalyst, and i) Faradaic efficiency for CO_2_RR and C_2+_ current density of ER‐CuNS catalyst at −1.45 V. Reproduced under a Creative Commons Attribution License 4.0 International License.^[^ ^66^ ^]^ Copyright 2022, The Authors.

The fundamental driving force of carbon spillover arises from adsorption energy differences between sites. When the adsorption strength of a metal site toward carbon species is weak, the species preferentially migrate to neighbouring sites with stronger adsorption, with the energy gradient constituting the primary impetus for migration. In the Ag‐Cu dual‐site catalytic system (**Figure** **6**a), Ag sites with weak CO adsorption (≈−0.25 eV) catalyse CO_2_ reduction to CO and promote its rapid desorption, generating a high local CO concentration, and the adjacent Cu sites with stronger CO adsorption (≈−0.75 eV) capture these CO molecules, forming a high‐coverage *CO intermediate state (Figure 6b).^[^ ^64^ ^]^

Beyond thermodynamic forces, migration kinetics are significantly influenced by interfacial electronic coupling. Electronic interactions between adjacent sites can modulate the activation barrier of migration transition state, while electron delocalization facilitates interfacial hopping of carbon species, accelerating migration. This effect is particularly pronounced at metal‐support or bimetallic interfaces. For example, Yang et al. designed a Cu‐Co dual single‐atom catalyst (CuCo‐DSAC), in which the spillover effect arises from the synergistic interplay between Cu and Co sites (Figure 6c). Electronic structure tuning and spatial proximity enable efficient CO_2_ reduction. As compared in Figure 6d,e, Co sites with a higher *d*‐band center, preferentially activate CO_2_ and stabilize *COOH intermediates, while the neighbouring Cu sites, with a lower *d*‐band center and electron‐deficient state, weaken CO adsorption and promote desorption. As a result, charge redistribution between the sites establishes a dynamic electronic buffer system, allowing rapid transfer of *COOH intermediates from Co to Cu, thereby preventing active site blockage and lowering reaction barriers.^[^ ^65^ ^]^

Local microenvironments further modulate migration behaviour. Factors such as surface electric fields, proton concentration, and solvation effects influence solvation states and migration barriers of carbon species. In electrocatalytic systems, applied potentials can regulate migration rates by altering the double‐layer structure. For instance, Ma et al. employed electrochemically reduced porous Cu nanosheets (ER‐CuNS) under strongly acidic electrolytes (pH ≤ 1) to achieve efficient CO_2_RR to C_2+_ products by synergistically tuning the local microenvironment (Figure 6f). Experimental and theoretical studies revealed that K^+^ accumulation at Helmholtz plane kinetically reduces proton coverage to suppress HER (Figure 6g,h), while thermodynamically stabilizing intermediates (e.g., *OCCO) to promote C‐C coupling, thereby enhancing C_2+_ selectivity and activity (Figure 6i).^[^ ^66^ ^]^ Additionally, the porous structure enriches K⁺ and OH^−^ through confinement effects for optimizing the local catalytic environment.

In summary, rational design of high‐performance catalysts and supports requires multi‐level synergistic optimization. Constructing multicomponent sites can effectively tune electronic structures, reduce migration barriers of carbon species, and enhance selectivity toward target products. Functionalized supports, such as reducible metal oxides (e.g., CeO_2_, TiO_2_), can facilitate adsorption and transport of carbon species via oxygen vacancies, while porous frameworks (e.g., MOFs, COFs) provide ordered channels and tunable chemical environments for directional migration and enrichment. Furthermore, precise interfacial engineering can optimize metal‐support interactions, modulate interfacial electronic structures and local microenvironments, thus constructing efficient carbon spillover pathways and achieving performance enhancement.

### 3.6 Intermediate Spillover

Intermediate spillover represents a primary dynamic behaviour in electrocatalytic processes, which are still under explorations, particularly regarding atomic‐scale migration mechanisms, dynamic interfacial regulation, and their coupling with realistic reaction environments. This phenomenon refers to the directional migration of reaction intermediates across a catalyst surface, which is crucial for efficient multi‐step catalytic reactions. After initial activation, intermediates should migrate across the surface to reach appropriate active sites for subsequent transformations, completing catalytic cycle. Such mechanisms are particularly significant in complex multi‐step reactions, such as NRR and MOR, where their effective implementation often dictates reaction rates and product selectivity.^[^ ^67^, ^68^, ^69^ ^]^

**Figure 7:**
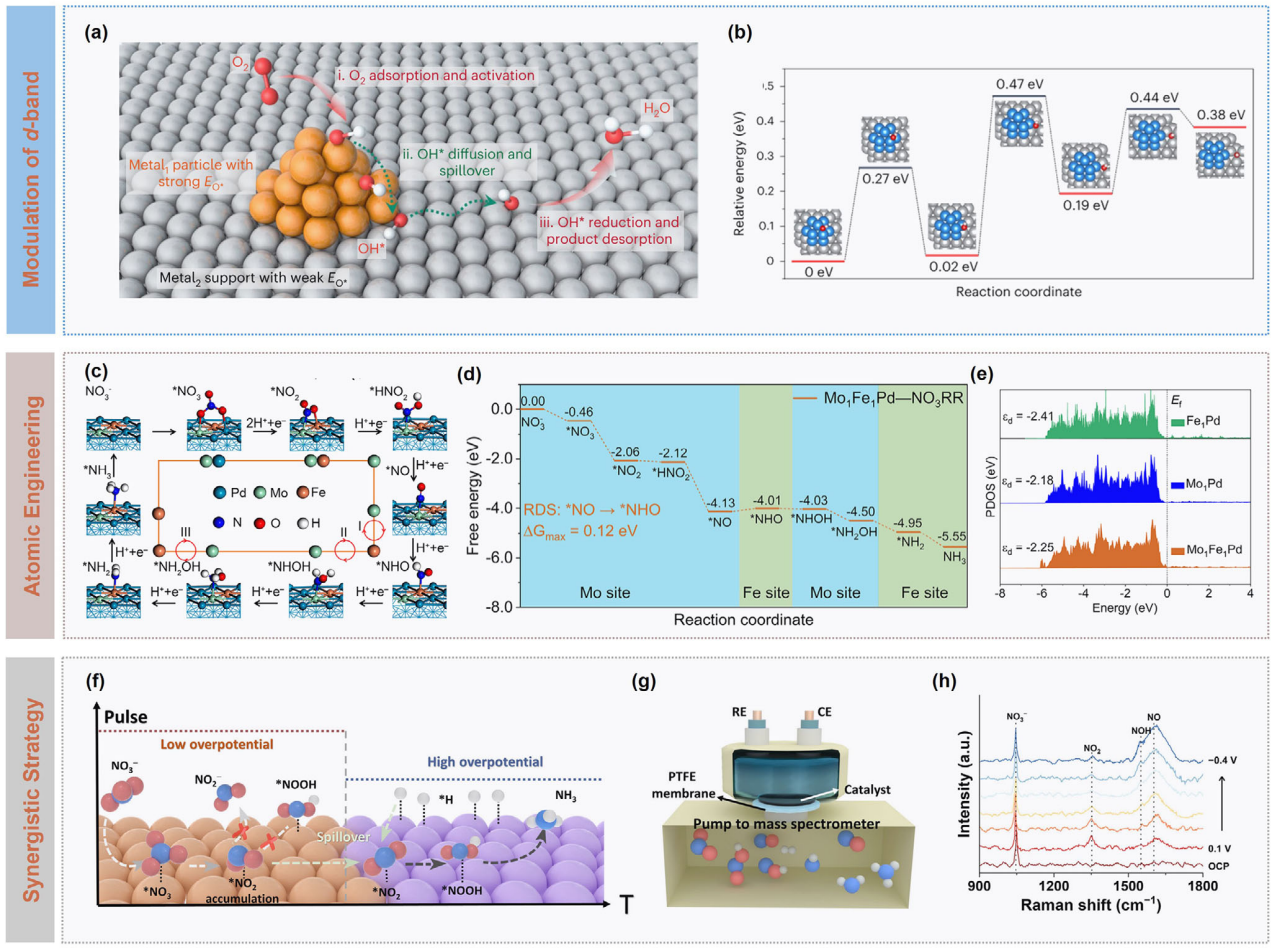
Catalytic dynamics modulation on intermediate spillover. a) Schematic illustration of the OH*‐spillover‐mediated ORR pathway, and b) Energy barriers for OH* diffusion across Pd‐Ag interface. Reproduced with permission.^[^ ^31^ ^]^ Copyright 2025, Springer Nature. c) Schematic illustration of reaction pathways of NO_3_RR on Mo_1_Fe_1_Pd, and d) Gibbs free energy and e) PDOS plots of Mo_1_Fe_1_Pd catalysts. Reproduced with permission.^[^ ^69^ ^]^ Copyright 2025, Wiley. f) Schematic illustration of structural merits of Cu@Co/NC and g) online differential electrochemical mass spectrometry (DEMS) technique, and h) in situ Raman spectra during NO_3_RR. Reproduced with permission.^[^ ^68^ ^]^ Copyright 2025, Wiley.

For multi‐electron electrocatalytic reactions, conventional catalysts are constrained by single active sites with fixed adsorption‐energy correlations for multiple intermediates, thus limiting their theoretical catalytic activity. Intrinsic linear scaling relationships between rection intermediates impose thermodynamic constraints, making overpotential reduction more challenging. Traditional single‐component catalysts are difficult to achieve simultaneously optimization on the adsorption behaviour of multiple intermediates, resulting in a performance bottleneck. To overcome the limitation, Wang et al. proposed an intermediate‐spillover strategy by constructing Pd/Ni(OH)_2_ and Pd/Ag dual‐component interfaces for ORR, as illustrated in **Figure** **7**a, in which Pd sites with strong adsorption characteristics activated O_2_ and generated OH intermediates, while Ni(OH)_2_ or Ag sites spontaneously received the spilled‐over OH and facilitated subsequent reduction and desorption.^[^ ^31^ ^]^ This mechanism enabled the spontaneous migration of OH* species from Pd to Ni(OH)_2_ for effectively circumventing the intrinsic scaling relationship between OOH* and OH* adsorption. Theoretical calculations simulations further revealed that such interfacial OH* transfer decoupled the multi‐step reaction across distinct active centers for independent optimization of the adsorption energies for each elementary step. Specifically, the diffusion barriers of OH* on Pd and Ag surfaces were as low as 0.45 and 0.27 eV (Figure 7b), respectively, while the barrier for cross‐interface spillover was nearly negligible, demonstrating the kinetic feasibility of this process.

Atomic‐precision catalyst design enables fine control of spillover pathways. In a Mo_1_Fe_1_Pd dual single‐atom alloy system, atomically dispersed Mo and Fe sites exhibit unique synergistic effects, promoting dynamic migration of intermediates between the two sites. This allows each elementary step in a multi‐step reaction to proceed along an optimized low‐barrier pathway, substantially enhancing catalytic efficiency. For example, the optimal pathway for NO_3_RR on Mo_1_Fe_1_Pd surfaces involves three successive intermediate migrations between Mo and Fe sites (Figure 7c), with the critical *NO to *NHO step exhibiting a barrier of only 0.12 eV (Figure 7d), representing reductions of 88% and 87% relative to single Mo (1.03 eV) and Fe (0.89 eV) sites, respectively. Electronic structure analysis indicates that dual single‐atom doping tunes the *d*‐band center to −2.25 eV (Figure 7e), enhancing adsorption of key intermediates while avoiding over binding at single sites, effectively circumventing conventional linear scaling constraints.^[^ ^69^ ^]^ Li et al.^[^ ^68^ ^]^ designed a Janus‐type Cu@Co bimetallic catalyst and combined it with a pulsed potential strategy (Figure 7f), for achieving atomistic and electronic‐level synergistic regulation of active site function and hydrogen supply in efficient nitrate‐to‐ammonia conversion. Specifically, Cu preferentially activated nitrate at low overpotentials to form nitrite intermediates, while Co acted as an effective hydrogen source at higher overpotentials to generate the key *NOOH intermediate. The dual‐potential pulsing (0.1 and −0.3 V vs RHE) mitigated the potential mismatch between hydrogen supply and intermediate hydrogenation, suppressing HER side reaction. Combined in situ techniques (Figure 7g,h) and theoretical calculations confirmed that *NOOH formation was the RDS, and that *NO intermediates migrated from Cu to Co, avoiding nitrite accumulation and providing mechanistic and experimental support for highly selective ammonia synthesis.

To summarize, these catalytic mechanisms leverage multi‐component synergy and directional intermediate spillover to overcome traditional reliance on single‐site adsorption energies, enabling precise control over primary reaction barriers and effectively suppressing side reactions. Future research for extending intermediate‐spillover strategies to broader catalytic transformations, such as C─C coupling, to bridge atomic‐level mechanisms with device‐scale performance, is a promising direction. Besides, in‐depth understanding of intermediate spillover and migration mechanisms is essential for the rational design of high‐performance electrocatalysts.

## 4 Functional Mechanisms of Spillover

### 4.1 Physical Spillover

**Figure 8:**
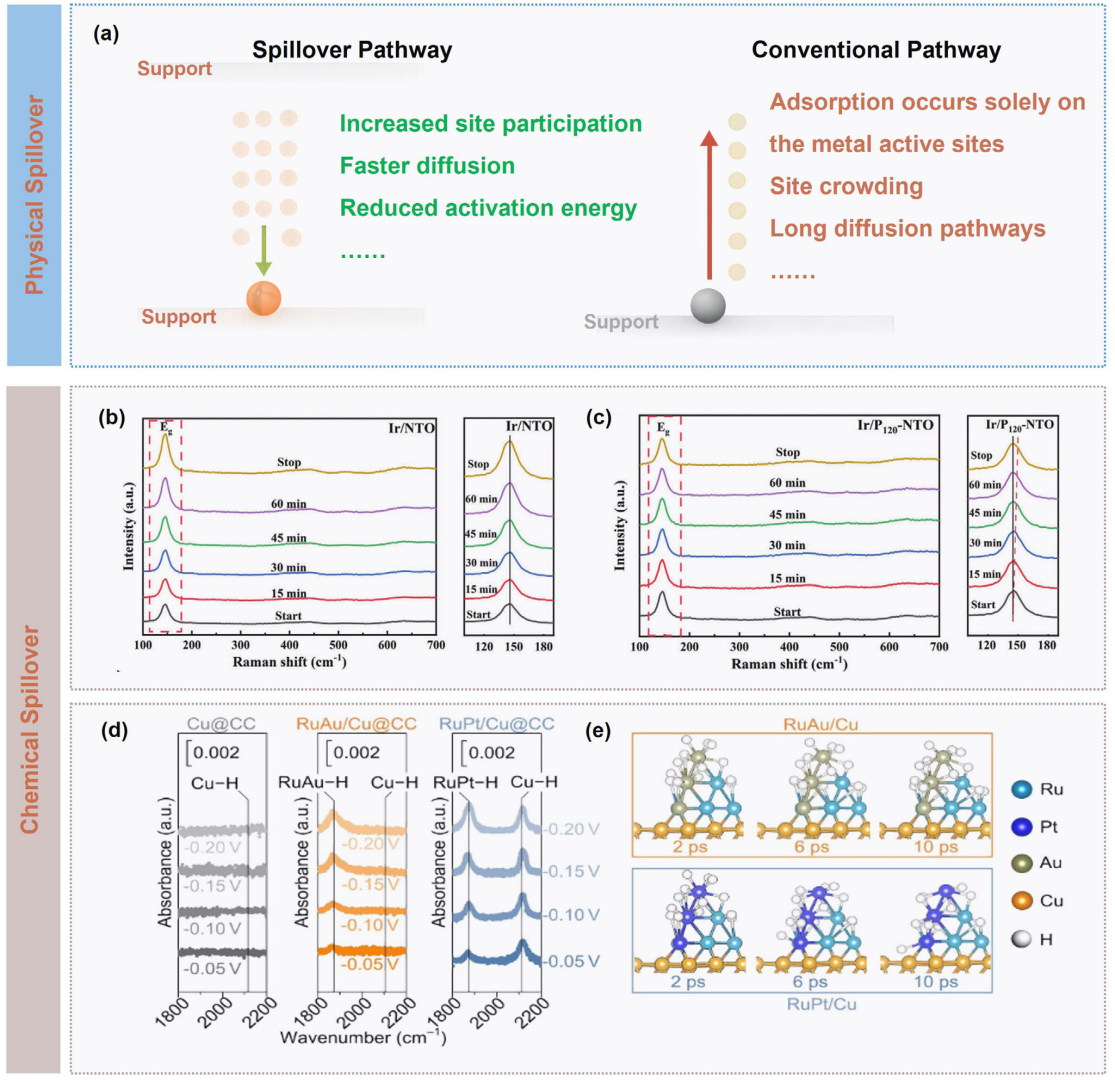
Functional mechanisms of physical and chemical spillover. a) Schematic illustration of spillover pathway and conventional pathway. b,c) Raman spectra of Ir/NTO and Ir/P_120_‐NTO at 1.5 V (versus RHE) during different OER times. Reproduced with permission.^[^ ^49^ ^]^ Copyright 2025, Wiley. d) Operando ATR‐IR spectra for the Cu@CC, RuAu/Cu@CC, and RuPt/Cu@CC catalysts at a potential range from −0.05 to −0.20 V (vs RHE) in the alkaline solution with and without nitrate addition, and e) adsorption behaviors for H* on the RuAu/Cu and RuPt/Cu models. Reproduced with permission.^[^ ^77^ ^]^ Copyright 2024, Wiley.

In conventional catalysis, as illustrated in **Figure** **8**a, active species are typically adsorbed and transformed solely at metal sites, which can lead to site crowding and require relatively long diffusion paths, thus limiting overall reaction rate. Physical spillover has emerged as an effective strategy to overcome these limitations. It refers to the migration of active species (e.g., H, or O) across the support surface primarily via physical adsorption interactions, such as *van der Waals* forces or electrostatic interactions, without forming strong chemical bonds with the support or undergoing significant electron transfer. This process is predominantly dominated by physical properties of the support, including specific surface area, pore architecture, and surface defects, which collectively influence transport efficiency of spilled‐over species. Such a mechanism not only expands the number of accessible active sites but also accelerates mass transport and lowers reaction barriers, thereby significantly enhancing catalytic performance. For example, Li et al. constructed a catalyst with Pd single atoms and sub‐nanometre clusters co‐anchored on carbon‐nitride nanosheets (Pd/C_3_N_4_), in which hydrogen molecules first dissociated at Pd cluster sites and subsequently migrate via physical spillover to neighbouring Pd single‐atom sites, while cinnamaldehyde molecules preferentially adsorbed on single‐atom sites to undergo hydrogenation.^[^ ^71^ ^]^ Aberration‐corrected electron microscopy and X‐ray absorption fine structure spectroscopy directly confirmed the coexistence of Pd single atoms and clusters, and theoretical calculations elucidated energy landscape of hydrogen spillover and associated reaction activation mechanism.

### 4.2 Chemical Spillover

Chemical spillover describes the dynamic process in which active species (e.g., H or O) form chemical bonds, undergo electron transfer, or participate in redox reactions with the support during migration, often significantly altering local chemical state of the support.^[^ ^72^ ^]^ This process typically relies on defects in the support, particularly oxygen vacancies, and can induce electronic rearrangement that affects the overall electronic structure of the catalyst.^[^ ^73^ ^]^ The pathways and extent of chemical spillover are governed by multiple factors, among which the intrinsic redox properties of the support are critical. Reducible oxides, such as CeO_2_, TiO_2_, and Fe_2_O_3_, readily facilitate chemical spillover due to their flexible valence states.^[^ ^74^, ^75^ ^]^ Meanwhile, strong metal‐support interactions can precisely modulate bonding strength of H/O species, guiding the spillover direction, whereas the concentration of defect sites can control the flux and reactivity of migrating species.^[^ ^76^ ^]^

In studies of oxygen spillover, Zhu et al.^[^ ^49^ ^]^ employed plasma treatment to precisely engineer interfacial oxygen vacancies in Ir/Nb‐TiO_2_ catalysts to establish directional OH^−^ transport channels from Ir active sites through oxygen vacancy regions to TiO_2_ support. The introduction of interfacial vacancies lowered the Ir *d*‐band center by 0.15 eV for weakening Ir‐OH binding and reducing the OH^−^ migration barrier from 0.95 to 0.46 eV, thereby substantially enhancing intermediate transport efficiency. In situ Raman spectroscopy (**Figure** **8**b,c) captured dynamic migration of OH^−^ from Ir to TiO_2_, confirming the determining role of oxygen vacancies as intermediate carriers. Specifically, the changes on E*_g_* mode corresponding to O‐Ti‐O symmetric stretching revealed a blue shift and peak intensity attenuation in Ir/P_120_‐NTO, which ceased upon reaction termination, whereas the E*_g_* peak of Ir/NTO remained stable, indicating negligible OH adsorption on the surface. Hence, the concurrent decay of the Ir‐ OH_ad_ signal and the blue shift of O‐Ti‐O vibrations collectively demonstrated accelerated desorption of OH_ad_ from Ir nanoparticles and directional migration to oxygen vacancies in P_120_‐NTO.

In hydrogen spillover systems, similar chemisorption mechanisms are commonly observed. Li et al. identified Cu‐H vibrational signals via in situ ATR‐IR (Figure 8d), confirming chemical adsorption of H on Cu surfaces in RuM/Cu (M = Pt, Ir, Rh, and Au) catalysts.^[^ ^77^ ^]^ Differential charge density analysis indicated that interfacial electronic delocalization underlies the weakened H adsorption, while theoretical simulations dynamically captured bond reorganization of H* during cross‐interface migration (Figure 8e).

In summary, physical and chemical spillover constitute two complementary mechanisms for interfacial species transport. Physical spillover, mediated by intermolecular interactions, enables mild migration with broad applicability and minimal structural perturbation but offers limited transport efficiency and selectivity. Chemical spillover, in contrast, achieves highly efficient and directional transport via chemical bonding, suitable for precise control of reaction pathways, albeit with higher activation barriers and potential surface reconstruction. In practical catalysis, these mechanisms are often operated synergistically. For instance, Bai et al. demonstrated a Pt/s‐MoO_3_ system that dissociated H from Pt could rapidly diffuse through a 3D interconnected branched network (physical spillover) while simultaneously forming stable H(O)*_x_* MoO_3_ intermediates with Mo^5+^ (chemical spillover).^[^ ^78^ ^]^ The 3D branched structure could provide efficient mass transport channels, and the porous surface could offer abundant active sites, and the step edges and oxygen vacancies could facilitate electron transfer and H stabilization. It was further revealed that ideal spillover supports should integrate physical transport advantages with controllable chemical active sites.

## 5 Dual Characteristics of Spillover

### 5.1 Advantages of Spillover

Spillover effects create dynamic transport channels between metal and support, enabling efficient dissociation and directional migration of active species. This not only significantly enhances catalytic activity but also allows precise control over reaction selectivity. Compared to conventional catalytic systems, spillover effectively mitigates reactant accumulation and mass transport limitations arising from uneven active site distribution, thereby suppressing side reactions and improving overall catalytic efficiency.

**Figure 9:**
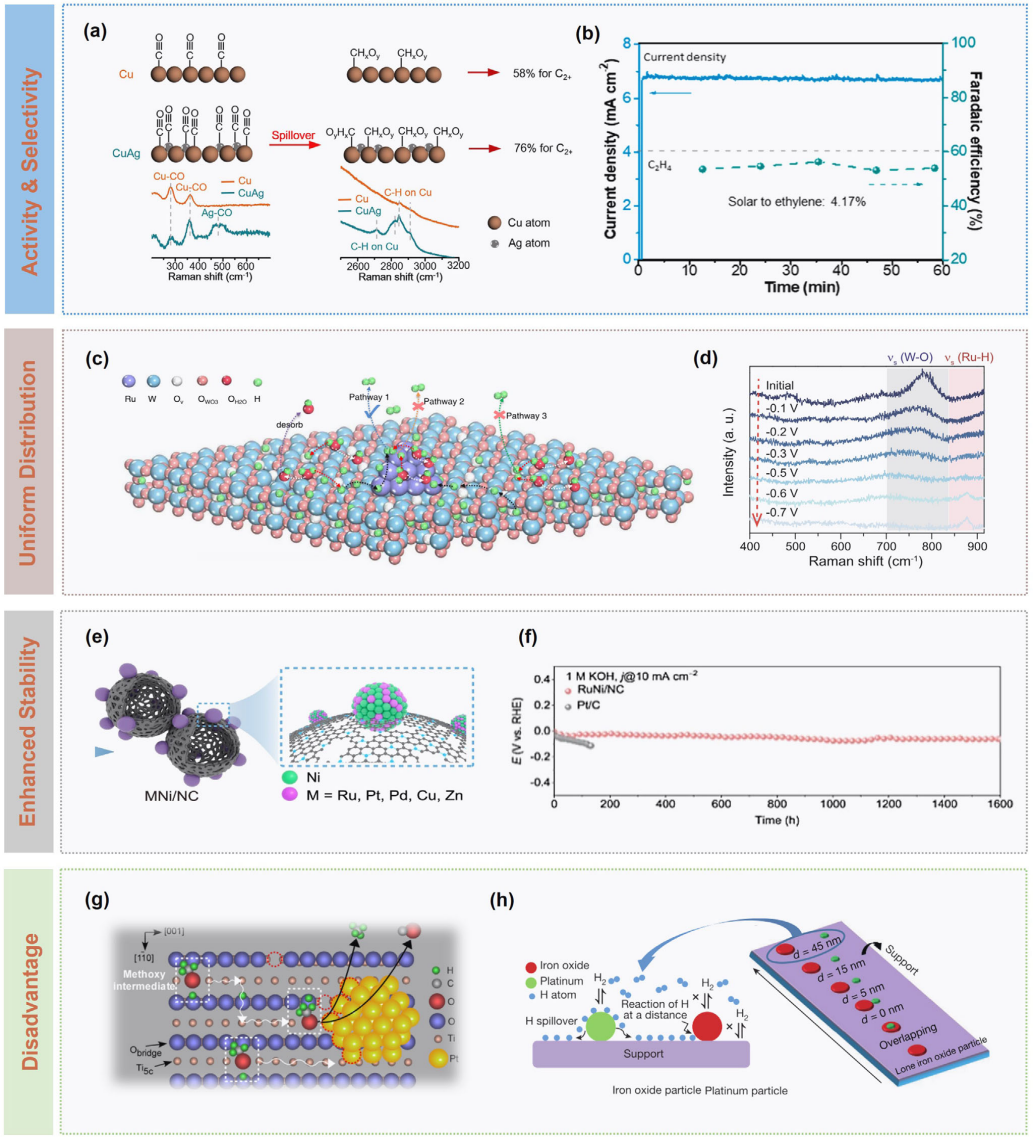
Crucial characteristics of spillover effects. a) Structural and chemical characterization of Cu_2_O and Ag‐decorated Cu_2_O (Cu_2_O‐Ag), and b) plots on current density and Faradaic efficiency of ethylene over reaction time. Reproduced with permission.^[^ ^27^ ^]^ Copyright 2019, American Chemical Society. c) Schematic illustration of hydrogen spillover, and d) in situ Raman spectra of Ru‐WO_3‐_ *_x_* catalyst /CP recorded in 1.0 m PBS solution. Reproduced under a Creative Commons Attribution License 4.0 International License.^[^ ^81^ ^]^ Copyright 2022, The Authors. e) Schematic illustration of synthesis and structure of MNi/NC (M=Ru, Pt, Pd, Cu, Zn) catalysts, and f) chronoamperometry tests of RuNi/NC at 10 mA cm^‒2^ in 1 m KOH. Reproduced with permission.^[82]^ Copyright 2024, Springer Nature. g) Schematic illustration of intermediate adsorbed on a TiO_2_ (110) surface. Reproduced with permission.^[^ ^70^ ^]^ Copyright 2023, American Chemical Society. h) Schematic illustration of hydrogen spillover on the aluminum oxide support. Reproduced with permission.^[^ ^26^ ^]^ Copyright 2017, Springer Nature.

Gao et al. reported a Cu‐Ag bimetallic catalyst that ≈95% of CO generated on Ag sites could be transferred to adjacent Cu sites via dynamic desorption‐re‐adsorption or interfacial diffusion.^[^ ^27^ ^]^ This highly efficient intermediate transfer not only reduces CO byproduct formation but also supplies sufficient *CO for C─C coupling reactions. By constructing a mesoscopic dynamic catalytic microenvironment composed of Cu_2_O nanowires and uniformly dispersed Ag nanoparticles, the surface residence time of CO intermediates were extended and spatial confinement was employed to suppress methane and other side reactions (**Figure** **9**a). This Ag generation‐CO spillover‐Cu coupling mechanism resulted in a solar‐driven CO_2_‐to‐ethylene conversion efficiency of 4.2% (Figure 9b). In oxygen spillover systems, Shen et al. developed a H‐Pt‐W_3_O/WC catalyst that leveraged lattice oxygen migration to overcome limitations in hydrogen oxidation reaction (HOR) activity and CO tolerance.^[^ ^79^ ^]^ Experimental results confirmed that lattice oxygen from W_3_O dynamically migrated to Pt sites, forming active Pt─O bonds. Also, synchrotron and isotope‐labelling studies revealed that spillover oxygen directly oxidized CO adsorbed on Pt, while Lewis acid sites on the support provided a secondary removal pathway via *OH capture. In addition, theoretical calculations further indicated that electron transfer from the support to Pt lowered the *d*‐band center, optimizing hydrogen adsorption while weakening CO binding strength.

Spillover also dynamically regulates the migration and distribution of active species on catalyst surfaces, enabling uniform reconstruction of active sites and synergistic optimization of reaction kinetics. It establishes efficient surface diffusion channels and precisely controls intermediate coverage and migration behaviour, which fundamentally alters the RDS of specific reactions.^[^ ^80^ ^]^ For example, Chen et al.^[^ ^81^ ^]^ exploited a Ru‐WO_3‐_ *_x_* catalyst to uncover a proton storage‐spillover mechanism (Figure 9c), in which oxygen‐deficient WO_3‐_ *_x_* acts as a proton reservoir for directionally transferring protons to Ru surface under cathodic potential to increase hydrogen coverage by 3–5 times. This was also verified through electrochemical impedance spectroscopy‐derived pseudo‐capacitance (Cφ) and in situ Raman monitoring of Ru‐H vibrational intensity (Figure 9d). Density functional theory (DFT) calculations showed that near‐ideal hydrogen adsorption free energy (∆G_H*_≈ 0) on Ru ensured near‐saturation hydrogen coverage (θ_H_ ≈ 1) at working potentials, and the reduced proton migration barrier shifted the RDS from water dissociation to hydrogen atom recombination. Recently, Feng et al. utilized electronic metal‐support interactions to induce hydrogen spillover, in which WS_2_ support enhanced the hydrogen adsorption of Pt for increasing surface concentration of hydrogen atoms at Pt active sites.^[^ ^82^ ^]^ This facilitated directional H migration from the metal to the support, greatly accelerating the HER kinetics. This dynamic balance optimizes HER kinetics in neutral media and explains how spillover modulates electronic structure to achieve performance enhancement.

Furthermore, spillover‐based dynamic networks are favourable for active species transport, improving both catalytic efficiency and system stability. Zhang et al. embedded RuNi nanoalloy tips into super‐hydrophilic carbon nanocages (Figure 9e), in which local electric fields generated by the tip effect enriched hydrated K^+^ and reconstructed the interfacial hydrogen‐bond network for optimizing water dissociation kinetics and intermediate adsorption behaviours.^[^ ^83^ ^]^ Experimental and theoretical results revealed that Ru catalysed water dissociation while Ni facilitated hydrogen recombination via spillover effects, forming an efficient Volmer‐Tafel relay pathway. As a results, the optimal catalyst manifested an overpotential of only 12 mV to achieve the density of 10 mA cm^−2^ and a stability for 1600 h (Figure 9f). Shen et al. constructed Ir‐Ru solid‐solution catalysts in low‐concentration alkaline electrolyte (0.05 m KOH), in which hydrogen spillover reduced *H coverage on Ru under high current, enhancing HER kinetics and stability.^[^ ^84^ ^]^ The resultant catalyst presented a cell voltage of only 1.75 V cell voltage at 1 A cm^−2^ in anion exchange membrane water electrolysis (AEMWE) and a stable operating time for over 1000 h. These studies provide atomic‐scale insights into hydrogen spillover mechanisms and open avenues for designing highly efficient and stable metal‐support catalysts.

### 5.2 Disadvantages of Spillover

Spillover effects in catalytic reactions exhibit a distinctive dual characteristic in the presence of both opportunities and challenges. On one hand, as mentioned above, spillover can significantly enhance catalytic activity and selectivity; on the other hand, uncontrolled migration of active species may induce side reactions. This negative effect is particularly pronounced in multistep complex reaction systems. When spillover species, such as H or O, exceed the capture capacity of target active sites, they may diffuse into non‐active regions and trigger undesired reactions. For instance, in the CO_2_RR process, excessive hydrogen spillover may promote the formation of by‐products such as CO, resulting in a decrease in selectivity.^[^ ^85^, ^86^ ^]^ Similarly, in HOR, although spillover facilitates hydrogen activation, overly mobile hydrogen in H‐rich environments can react with CO_2_ or surface carbon species, leading to undesired methane formation and significantly diminishing selectivity.^[^ ^87^, ^88^ ^]^ To address this issue, multiscale modulation strategies, including optimization of metal particle distribution, selection of support types, and design of metal‐support interfaces, have proven as effective strategies in suppressing side reactions.

For instance, Liu et al. elucidated the dynamic equilibrium mechanism of methanol catalysis over a Pt/TiO_2_ (110) system, showing that methoxy intermediates undergo spillover and reverse‐spillover between Pt and the TiO_2_ support, which significantly alter reaction pathways (Figure 9g). By tuning the density of Pt nanoparticles, the diffusion path of methoxy species were shortened, which could enable the rapid return to Pt sites for preferential dehydrogenation, thereby reducing CH_4_ selectivity from 77% to 55%.^[^ ^70^ ^]^ Karim et al.^[^ ^26^ ^]^ employed an electron‐beam lithography to construct Pt/iron oxide model systems with precisely controlled interparticle spacing (0–45 nm) and systematically compared the difference between reducible supports (TiO_2_) and non‐reducible ones (Al_2_O_3_) (Figure 9h). It was demonstrated that TiO_2_ enabled efficient long‐range spillover via proton‐electron cooperative transfer, allowing hydrogen activity to extend far from metal sites. In contrast, Al_2_O_3_, which lacked electronic conduction pathways and held three‐coordinated Al sites prone to competitive water adsorption, exhibited severely limited spillover efficiency and range. Also, the study highlighted that metal particle size and distribution should balance hydrogen dissociation and migration kinetics. Specifically, the smaller particles provided more active sites but might restrict effective hydrogen migration. On reducible supports, sparse metal dispersion ensured efficient spillover, whereas non‐reducible supports required denser particle arrangement. Additionally, the direct metal‐support contact avoided spillover bottlenecks, while indirect contact depended on support properties.

Finally, spillover inherently involves complex interfacial processes, and precise control remains challenging. Although tuning structures and properties of metal and/or support can partially improve spillover behaviour, the process exhibits strong nonlinearity and dynamic characteristics due to the synergistic effects of metal‐support electronic interactions, surface defect distribution, and reaction environment. For example, the smaller metal particles can increase site density but may hinder hydrogen migration due to excessive interfacial interaction; meanwhile, dynamic processes such as surface hydroxylation and evolving support reduction further enlarge gaps between experimental outcomes and theoretical predictions. Thus, it is necessary to gradually establish structure‐performance relationships by combining experiments with theory. To achieve a transition from qualitative understanding to quantitative control, an in‐depth atomic‐scale elucidation of migration mechanisms and activity regulation principles remains prerequisite.

## 6 Catalyst Systems for Spillover

### 6.1 Metal Catalysts

The efficiency of spillover effects depends not only on the properties of the support but also on the intrinsic characteristics of metal particles and their interactions with the support. Studies have shown that the physicochemical properties of different precious metals, such as Pt, Pd, and Ru, significantly influence hydrogen spillover behaviour.

Park et al. developed a Pt single‐atom/nanoparticle composite system (Pt SA/WO_3‐_ *_x_*), which exhibited a mass activity of 12.8 A mg^−1^ that were 16.3 times higher than commercial Pt/C and an overpotential reduction of 105 mV.^[^ ^89^ ^]^ This remarkable enhancement is primarily attributed to the Pt single‐atom sites for effectively shorten hydrogen diffusion path. Wang et al. systematically compared hydrogen adsorption behaviours of Ru, Pt, and Ni on super‐activated carbon (AX‐21) and templated carbon (TC) supports and verified that the order of hydrogen adsorption capacity across different carbon structures as Ru > Pt > Ni.^[^ ^90^ ^]^ Shao et al. demonstrated controlled loading of 2 nm noble metal nanoparticles (Pt, Ir, Ru, Rh) on a ZIF‐67 support, revealing a hydrogen spillover ability trend of Pt > Ir > Ru > Rh.^[^ ^91^ ^]^

Among various noble metal catalysts, Pt‐based materials stand out due to their nearly barrier‐free H_2_ dissociation and excellent electronic conductivity for maintaining structural stability across wide potential windows and harsh conditions. However, practical applications remain challenged by CO poisoning, high migration barriers, and high cost. Current research solutions mainly include alloying engineering, constructing core‐shell architectures, and exploiting single‐atom/cluster systems.^[^ ^92^, ^93^, ^94^ ^]^ For example, Wei et al. anchored Pt nanoclusters on oxygen‐rich TiO_2_ supports, achieving a synergistic effect of reverse charge transfer and enhanced hydrogen spillover, and elucidated the oxygen vacancy‐induced electron transfer mechanism.^[^ ^95^ ^]^ Chen et al. further refined this strategy by spatially controlling oxygen vacancy distribution in Pt/TiO_2_ catalysts, revealing through atomic‐resolution characterization the specific interfacial cooperation between neighbouring oxygen vacancies and Pt sites in formaldehyde oxidation.^[^ ^96^ ^]^ Besides, Sn doping activated the oxygen spillover capacity of TiO_2_ supports, and multiscale characterization captured CO‐induced reverse oxygen spillover, significantly enhancing low‐temperature CO oxidation activity.^[^ ^97^ ^]^ Li et al. developed a P‐doped Pt_3_Co/NC catalyst, in which precise tuning of the *d*‐band center and construction of electron‐rich phosphorus sites enabled efficient proton capture and short‐range hydrogen spillover.^[^ ^98^ ^]^ Dai et al. demonstrated that atomic‐scale hydrogen spillover was achieved in the single‐phase oxide (La_2_Sr_2_PtO_7+δ_) via a synergistic mechanism involving O‐site adsorption, La‐Pt bridge migration, and Pt‐site desorption.^[^ ^99^ ^]^

In contrast, Pd‐based materials, while slightly weaker in H_2_ dissociation, possess an optimal *d*‐band center that affords favourable hydrogen adsorption balance. In alkaline HER, ORR, and MOR, Pd catalysts often perform comparably or even surpass Pt, with superior anti‐poisoning properties and cost‐effectiveness. However, hydrogen migration in Pd‐based systems often relies on support‐mediated long‐range pathways, necessitating sophisticated interfacial engineering to achieve high efficiency.^[^ ^100^, ^101^, ^102^ ^]^ It is noteworthy that bimetallic systems generally exhibit pronounced synergistic effects. Yan et al. reported that PtPd alloys on CeO_2_ supports outperformed monometallic Pt or Pd catalysts, in which Pt/CeO_2_ suffered from high desorption barriers due to excessive hydrogen adsorption, whereas Pd/CeO_2_ suffered from insufficient active site efficiency.^[^ ^103^ ^]^ Alloying Pt and Pd not only suppresses nanoparticle aggregation but also optimizes interfacial hydrogen adsorption via electronic effects. DFT calculations indicate that the Gibbs free energy of hydrogen adsorption at the three‐phase interface is as low as 0.023 eV. Tan et al. combined theoretical calculations and experimental validation to screen high‐performance binary electrocatalysts for hydrogen spillover using work function difference and hydrogen adsorption energy as the key descriptors.^[^ ^104^ ^]^ Among these candidates, PtIr‐MoS_2_ exhibited superior performance to the commercial Pt/C even at low metal loadings. These findings demonstrate the advantages of bimetallic catalysts in overcoming intrinsic limitations of monometallic systems and highlight the potential of precise metal‐support interfacial engineering for designing efficient and stable water‐splitting catalysts.

Ru‐based catalysts offer superior H_2_ dissociation and manifest cost advantages, however, they often suffer from excessively strong hydrogen adsorption at isolated active sites, thus limiting catalytic performance. Recent studies have shown that constructing Ru‐based heterostructures enables directional electron transfer from Ru to the support, which can optimize H and OH adsorption energies and facilitate dynamic interfacial migration, thus transforming this limitation into a unique advantage in hydrogen spillover catalysis.^[^ ^105^ ^]^ In the Ru‐WO_3_ system, hydrogen migrated from WO_3_ to Ru during HER and spilled back during HOR, which established reversible bidirectional transfer.^[^ ^106^ ^]^ At the Ru@Mn_3_O_4_ interfaces, moderate OH adsorption at Ru─O─Mn sites accelerated alkaline Volmer step and alleviated OH poisoning.^[^ ^107^ ^]^ Besides, tuning oxygen vacancies or oxygen affinity in supports, as in the Ru/NiMoO_4‐_ *_x_* system, further enhanced spillover kinetics by lowering migration barriers and interfacial electric fields.^[^ ^108^ ^]^ Overall, Ru catalysts utilize interfacial electronic synergy to overcome excessive hydrogen adsorption for efficient dynamic H/OH transfer, which enhances catalytic activity and stability.

**Figure 10:**
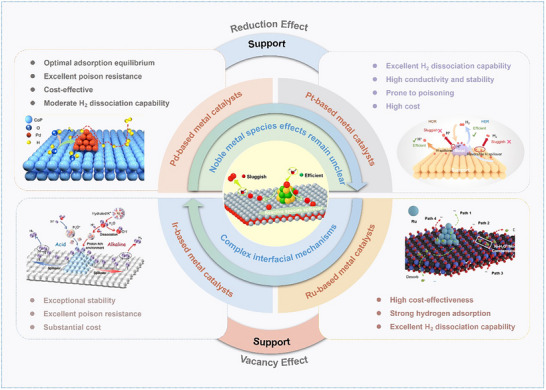
Schematic illustration of catalyst systems for spillover. Left: Reproduced with permission.^[^ ^102^ ^]^ Copyright 2024, American Chemical Society. Reproduced under a Creative Commons Attribution License 4.0 International License.^[^ ^138^ ^]^ Copyright 2024, The Authors. Right: Reproduced with permission.^[^ ^106^ ^]^ Copyright 2025, Wiley. Reproduced with permission.^[^ ^108^ ^]^ Copyright 2024, Wiley.

Despite the outstanding activity and efficiency of precious metal catalysts in spillover reactions, as summarized in **Figure** **10**, their high production cost remains a critical barrier for large‐scale applications. While rational structural designs can partially optimize spillover pathways and improve atomic utilization, a comprehensive evaluation of catalyst performance under realistic reaction conditions remains essential.

Beyond noble metals, non‐noble metal catalysts (e.g., Cu, Co, Ni, Mo) have emerged as promising candidates in electrochemical catalysis due to their low cost, abundant availability, and tunable properties. Strategies such as heterostructure construction, defect engineering, doping optimization, and interfacial electronic modulation allow precise tuning of active site electronic properties and adsorption behaviors, thus enabling efficient formation and migration of key intermediates. Multifunctional interfaces and synergistic support interactions further enhance electron and proton transport for optimizing the division of reaction steps and significantly improving overall catalytic performance. For example, Zheng et al. reported a CoNi‐LDH@Cu_2_O heterostructured catalyst that achieved enhanced nitrate electroreduction to ammonia via a synergistic hydrogen spillover and electron transfer mechanism. In this system, CoNi‐LDH could facilitate water dissociation to generate active hydrogen species, which subsequently migrated to the Cu_2_O interface through hydrogen spillover for accelerating hydrogenation. In the meanwhile, electron transfer from CoNi‐LDH to Cu_2_O could strengthen NO_3_^−^ adsorption and activation. This cooperative mechanism effectively lowers the water‐dissociation barrier and the rate‐determining step for NO‐to‐NOH conversion (0.43 eV), promoting an energetically favorable NOH‐mediated reduction pathway.^[^ ^109^ ^]^ Dessalew et al. reported a dual‐site Ni_3_Se_4_‐Ni_3_N heterostructure, in which Ni_3_N dissociated water to enrich surface hydrogen, and Ni_3_Se_4_ promoted H coupling and H_2_ release, and interfacial electronic transfer facilitated hydrogen migration from Ni_3_N to Se sites.^[^ ^110^ ^]^ Besides, Sun et al. reported a MoCo dual‐atom catalyst, in which oxygen‐vacancy‐induced internal electric fields could promote active hydrogen migration between Mo and Co sites. It was concluded that the local field generated by oxygen vacancies accelerates the diffusion of positively charged hydrogen atoms, enhancing the hydrogen evolution kinetics.^[^ ^111^ ^]^

### 6.2 Metal Oxide Supports

In spillover, supports can be broadly classified into reducible and non‐reducible types based on their capacity to store and transport oxygen species, which profoundly affects hydrogen migration pathways and reaction kinetics. Reducible supports, such as transition metal oxides with abundant oxygen vacancies and variable metal valence states (e.g., WO_3_ or TiO_2_), exhibit unique advantages in spillover catalysis. These materials not only serve as supports for active sites but also facilitate hydrogen adsorption, activation, and migration via their redox properties, significantly enhancing spillover effects. Nevertheless, some major challenges such as poor conductivity and structural instability under acidic conditions limit practical applications. In proton exchange membrane fuel cells and other strongly acidic environments, supports can dissolve or undergo phase transitions, causing loss of active species, disruption of interfacial structures, and interruption of hydrogen spillover pathways. In addition, the limited electronic conductivity weakens metal‐support synergy, reducing hydrogen transport and reaction kinetics.

To address these limitations, recent research has focused on interface engineering and atomic‐level structural modulation. Some typical strategies such as constructing heterojunctions, introducing carbon interlayers, generating vacancies, and/or designing core‐shell architectures, have effectively enhanced both conductivity and chemical stability of supports. For example, Wang et al. employed advanced in situ characterization to directly observe dynamic interactions between CeO_2_ supports and Ni active sites, revealing the evolution from Ni nanoparticles to single atoms and providing direct evidence for hydrogen spillover on CeO_2_.^[^ ^112^ ^]^ Notably, metal oxides often play dual roles in catalytic systems, as reducible supports promoting hydrogen spillover and as active sites for the reaction. Rajalakshmi et al. developed a WO_3_/graphene composite catalyst in which the synergistic interaction between hexagonal WO_3_ nanorods and graphene reduced reaction barriers to near‐Pt benchmark levels.^[^ ^113^ ^]^ Mechanistic studies indicated that graphene, with its sp^2^‐hybridized carbon network, provided excellent electronic conductivity and abundant active sites, outperforming other carbon supports (GR > rGO > GO > CB), and WO_3_ primarily served as an efficient hydrogen adsorption site, whereas graphene channels hydrogen migration and desorption, significantly enhancing overall reaction kinetics. Fu et al.^[^ ^114^ ^]^ constructed a CoO‐C‐SnO_2_ ternary composite to achieve a reverse hydrogen spillover effect, in which protons migrated from CoO, acting as a solid base, through a carbon channel to the surface of SnO_2_, a solid acid. This process significantly enhanced low‐temperature oxygen activation on SnO_2_, promoted *OOH intermediate formation, and lowered the energy barriers for C─H and C─C bond cleavage, thus improving activity and selectivity for ethanol oxidation. In addition to metal oxides, Zhao et al. reported a CoP@Ni_2_P heterostructured nanowire array by a controlled phosphorization, which tuned work function difference between dual‐metal sites, reduced interfacial hydrogen spillover barriers.^[^ ^115^ ^]^ It was unveiled that Ni_2_P sites facilitated water splitting and hydrogen generation, while CoP sites efficiently adsorbed nitrate, enabling interface‐mediated hydrogen spillover to promote nitrate reduction via an indirect pathway toward ammonia synthesis.

Hydrogen spillover is not restricted to conventional reducible oxides, but also occurs on non‐reducible supports, such as SiO_2_, Fe_2_O_3_, and Al_2_O_3_. For instance, a CoO*_x_*/Al_2_O_3_/Pt model system was constructed using atomic layer deposition (ALD), and hydrogen spillover was optimized by tuning Al_2_O_3_ interlayer thickness (5–41 nm).^[^ ^116^ ^]^ In situ characterizations revealed the hydrogen‐induced reduction of CoO_x_ and dynamic modulation of electronic structure, leading to significantly enhanced catalytic activity. Bai et al. realized a long‐range (>50 nm) hydrogen spillover in non‐reducible MOFs.^[^ ^30^ ^]^ By tuning ligand functional groups (e.g., ─CHO, ─OH, ─NH_2_) or introducing water molecules, hydrogen migration was substantially promoted while maintaining structural integrity of MOFs. Using a sandwich‐structured MOFs@Pt@MOFs catalyst, a highly selective hydrogenation of N‐heteroaromatics was achieved, highlighting the controllability and practical potential of hydrogen spillover in catalytic reactions. Li et al. systematically investigated Pt‐induced hydrogen spillover dynamics on the monoclinic γ‐WO_3_ surface.^[^ ^117^ ^]^ At room temperature, Pt clusters facilitated hydrogen spillover for generating W^5+^ species and hydrogen intermediates. With increasing temperature, competitive processes including water desorption, reverse hydrogen spillover, and hydrogen diffusion from the surface into the bulk resulted in sequential reoxidation and reduction of near‐surface tungsten atoms.

### 6.3 Interfacial Spillover

The precise engineering of active component‐support interfaces plays a crucial role in spillover reactions. These processes rely on the migration of active species from strong adsorption sites to weaker adsorption sites, which often overcome substantial interfacial energy barriers. To reduce the spillover barriers, various strategies at the atomic and electronic levels have been proposed to achieve precise control over interfacial reaction pathways.^[^ ^118^ ^]^

As mentioned above, the work function difference between metals and compounds is a key factor governing interfacial electronic structures and dominating spillover kinetics. By optimizing work function alignment, interfacial charge accumulation can be suppressed for significantly lowering migration barriers. Based on this principle, strategies such as alloying, ligand modification, defect engineering, and heteroatom doping are widely applied to regulate interfacial electronic structures. Specifically, alloying can adjust the *d*‐band electronic structure of metals to align the Fermi level with that of the support for reducing Schottky barriers and charge accumulation and providing low‐resistance pathways for hydrogen migration.^[^ ^103^, ^105^ ^]^ Ligand modification can alter the local environment of active sites through electronic and steric effects to modulate H intermediate adsorption and provide transient hydrogen‐bonding sites for reducing the barriers for H dissociation and migration. For example, Li et al. introduced ethylene glycol ligands at the Pt/CoP interface to construct a composite interface that simultaneously enriched protons and modulated electronic properties, which effectively lowered the hydrogen migration barrier from Pt to CoP and achieved HER activity beyond commercial Pt/C while improving precious metal utilization.^[^ ^119^ ^]^ Defect engineering introduces structural defects such as oxygen vacancies at interfaces to form efficient electron‐donating centers that induce charge redistribution between metals and supports. This weakens metal‐hydrogen binding and establishes favorable electronic channels for hydrogen transport to synergistically optimize desorption and migration. Niu et al. constructed Ru/ac‐ZrO_2_ catalysts in which oxygen vacancy‐induced electron transfer significantly enhanced alkaline HER performance, and the presence of vacancies lowered the ZrO_2_ work function, thus promoting electron transfer to Ru and accelerating hydrogen spillover.^[^ ^120^ ^]^ Heteroatom doping can tune the host material's band structure and work function to better match the metal component to lower hydrogen migration barriers. Zhu et al. used F‐doping to modulate electronic structure of Ru/F‐FeCoOOH heterostructures, which could reduce the work function difference to 0.05 eV, and weaken interfacial trapping of H intermediates and promoting hydrogen spillover.^[^ ^121^ ^]^

Beyond conventional metal‐support interfaces, other interface types can effectively drive spillover. In compound‐compound interfaces, the built‐in polarization fields formed at heterojunctions can serve as intrinsic driving forces to promote migration from strongly adsorbing to weakly adsorbing phases and optimize reaction pathways. For instance, Jiang et al. revealed that a NiSe_2_‐Ni_5_P_4_ heterojunction with a small work function difference (0.08 eV) reduced interfacial charge accumulation and weakened H intermediate adsorption, thus lowering the H migration barrier from NiSe_2_ to Ni_5_P_4_ to 0.40 eV. Also, NiSe_2_ served as the water dissociation site that facilitated the Volmer step, while Ni_5_P_4_ functioned as the hydrogen desorption site that completed the Tafel step, which established a thermodynamically spontaneous and kinetically efficient hydrogen spillover pathway that delivered excellent HER performance.^[^ ^122^ ^]^ At compound‐metal interfaces, a reverse spillover mechanism was proposed, in which the compound acted as a “hydrogen reservoir” delivering activated H controllably to metal sites for enhancing surface H coverage and lowering dissociation barriers. Su et al. constructed Ni/MoBT*_x_* heterostructures via dual‐site interface engineering to achieve a “reaction step decoupling” mechanism, in which MoBT*_x_* catalyzed water dissociation to produce adsorbed H, which migrated to Ni sites via reverse spillover, and Ni with an optimized electronic structure served as the ideal desorption center for balancing Volmer and Tafel adsorption requirements and improving alkaline HER.^[^ ^123^ ^]^ In metal‐metal or semimetallic interfaces, spillover is primarily governed by differences in *d*‐band centers due to the absence of pronounced Schottky barriers.^[^ ^124^ ^]^ Deng et al. reported Pd nanoparticles/Ir metalene heterojunction catalysts, which exhibited excellent HER performance in acidic media. Pd sites provided strong H adsorption, while Ir metalene facilitated H desorption, and the small Fermi level difference (0.76 eV) promoted H migration from Pd to Ir to shift the rate‐determining step from Volmer to Tafel. Also, it was demonstrated that hydrogen spillover lowered ΔG‐H_ads_ on Pd sites (−0.19 eV), optimized adsorption–desorption equilibrium, and modulated *d*‐band centers via interfacial charge redistribution, which further enhanced catalytic activity.^[^ ^125^ ^]^

Overall, interface‐focused spillover research reveals distinct behaviors and intrinsic synergies across different interface types. Strategies such as work function alignment, defect regulation, and heteroatom doping achieve energy‐level matching and electronic reconstruction, which effectively lowers migration barriers and optimizing adsorption. The cooperative action of multiple interfaces facilitates spatial and energetic partitioning of water dissociation, hydrogen adsorption, and desorption, which substantially improves overall catalytic efficiency. With an improved understanding of interfacial electronic structures and reaction kinetics, these advances provide a theoretical foundation for the rational design of efficient, stable and economically viable spillover catalytic systems.

### 6.4 Industrial Consideration

To transform these electrocatalytic systems from laboratory demonstrations to device‐level or even industrial applications represents a critical step in translating nanoscale high‐efficiency reaction mechanisms into macroscopically feasible energy conversion processes. Cooperative catalytic strategies based on spillover effects have shown remarkable potentials. For instance, Zhu et al.^[^ ^49^ ^]^ constructed oxygen‐vacancy‐rich interfaces between metallic Ir and Nb‐doped TiO_2_O_2_O_4_, which enabled oxygen spillover from Ir to the support. When applied as an anode catalyst in proton exchange membrane water electrolysis (PEMWE), the optimal catalyst delivered a device efficiency of 72.2% at 1 A·cm^−2^, with an energy consumption of 4.07 kW·m^−3^ H_2_ (≈45.3 kWh·kg^−1^). Moreover, the voltage decay rate remained as low as ≈40 µV·h^−1^ under continuous operation at 1.0 A·cm^−2^ and 65 °C for 500 h. Fang et al.^[^ ^126^ ^]^ designed an IrO*_x_*/t‐ZrO_2_ anode catalyst with excellent PEMWE performance and long‐term stability. The t‐ZrO_2_ support, which was rich in oxygen vacancies and possessed high oxygen‐ion conductivity, could promote the spillover migration of reactive oxygen species between IrO*_x_* and the support. This could mitigate local oxygen accumulation and structural stress for preserving lattice integrity and catalytic activity of IrO*_x_*. Experimental measurements concluded that the resulting catalyst showed a current density of 3.10 A·cm^−2^ and a mass activity of 31000 A·g Ir^−1^ at 80 °C and 1.9 V. Contributed by the spillover channels for facilitating timely removal of reactive oxygen intermediates to prevent over‐oxidation and dissolution of Ir sites, a voltage decay rate of only ≈6.25 µV·h^−1^ and an Ir loss of ≈1% after 1 A·cm^−2^ were achieved after operation for 1600 h.

Despite these advances, the industrial‐scale translation faces substantial challenges. First, the controllable scaling of the catalytic layer is important. Specifically, nanoscale interfaces achieved in the laboratory are difficult to maintain uniformly over large‐area electrodes, and microstructural heterogeneity can diminish spillover effects and product selectivity.^[^ ^127^ ^]^ Second, multi‐scale mass transport optimization remains a bottleneck in electrolyzer design. At high current densities, gas supply, ion migration, and product removal should achieve dynamic balance within the porous electrodes. Otherwise, the local concentration gradients, potential heterogeneity, or bubble accumulation can trigger side reactions and performance decay. Flow field and thermal management uniformity directly affect system stability, as local overheating or potential gradients may induce catalyst phase transitions or support degradation, potentially causing system failure. Currently, in terms of water splitting devises, proton exchange membrane (PEM) and anion exchange membrane (AEM) technologies represent complementary pathways. In general, PEM operates under acidic conditions, which relays on noble metal catalysts but providing high current density and energy efficiency. AEM allows the use of non‐noble catalysts with cost advantages, though its ionic conductivity and chemical stability remain limited.^[^ ^128^ ^]^ Finally, the long‐term durability is another challenge for commercialization. Spillover‐dependent heterointerfaces are prone to some dynamic processes, such as Ostwald ripening, elemental interdiffusion, or chemical‐state reconstruction, thus leading to the deactivation of cooperative mechanisms. Also, the existing electrode‐pore flooding, product‐salt crystallization, membrane degradation, and product crossover might be magnified during scale‐up, leading to the obvious performance decay.^[^ ^129^ ^]^

In summary, the opportunities in electrocatalytic scale‐up lie in achieving synergistic breakthroughs across material innovation, interface engineering, and system integration. Future progress requires overcoming multi‐scale challenges from nanoscale structure control to reactor system optimization to realize electrocatalytic systems combining high activity, stability, and scalability. It is expected that advanced catalytic concepts such as spillover effects can be transformed from the laboratory to industrial production through the in‐depth integration of interdisciplinary cooperation including materials science, electrochemistry, energy and chemical engineering, which provides sustainable solutions for clean energy conversion and carbon‐neutral technologies.

## 7 Spillover‐Mediated Catalytic Reactions

### 7.1 HER

**Figure 11:**
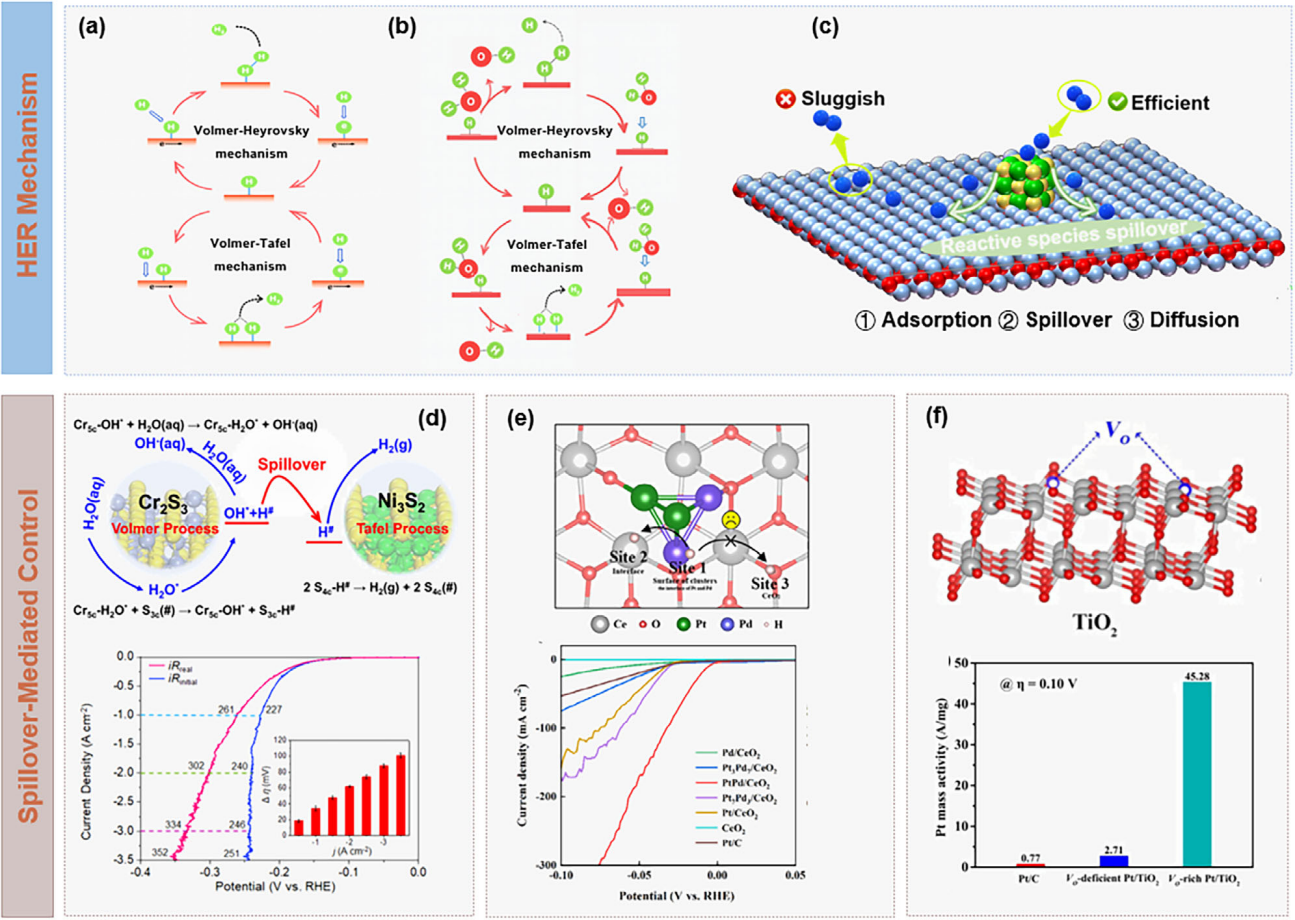
Spillover‐mediated mechanisms in HER. a–c) Schematic illustration of traditional non‐spillover catalyst reactions: (a) acidic HER, (b) non‐acidic HER, and (c) hydrogen spillover migration path. d) H* migrates from Cr_5c_ sites on Cr_2_S_3_ to Ni_3_S_2_ to generate H_2_, and LSV curves. Reproduced with permission.^[^ ^133^ ^]^ Copyright 2022, American Chemical Society. e) H* migrates from PtPd to the CeO_2_ interface, achieving near‐zero hydrogen adsorption free energy, and LSV curves. Reproduced with permission.^[^ ^103^ ^]^ Copyright 2025, Wiley. f) Pt nanoclusters on VO‐PtTiO_2_O_4_ show enhanced activity via electron‐rich Pt and H* spillover to TiO_2_, and the Pt mass activity. Reproduced with permission.^[^ ^95^ ^]^ Copyright 2021, Wiley.

To uncover the mechanistic role of hydrogen spillover in HER, it is essential to recognize the fundamental nature of HER as a prototypical two‐electron transfer process. As a half‐reaction in electrocatalytic water splitting and proton reduction, HER pathway is synergistically governed by the intrinsic properties of the catalyst and the external reaction environment.^[^ ^130^ ^]^ In acidic media, HER typically proceeds via three critical steps (**Figure** **11**a). Initially, protons traverse the electrical double layer under electrode potential and adsorb electrochemically on catalytic active site (M), forming a surface hydrogen intermediate (M‐H*). This Volmer step (Equation (1)) is generally considered as the RDS. Subsequently, the adsorbed hydrogen species (M‐H*) desorb via two competitive pathways: the Heyrovsky step (Equation (2)), in which M‐H* undergoes electrochemical coupling with a proton from solution, and the Tafel step (Equation (3)), involving chemical recombination of two M‐H* species. The relative contribution of each pathway depends on surface hydrogen coverage, electrode potential, and the structure of the interfacial double layer.^[^ ^131^ ^]^

(1) $$Volmer\left(adsorption\right):H^{+}+e^{-}+M\to M-H^{*}\left(RDS\right)$$

(2) $$\begin{array}{ccc} Heyrovsky\left(electrochemicaldesorption\right): & & M-H^{*}+H^{+}+e^{-}\\ & & \;\to H_{2}+M \end{array}$$

(3) $$Tafel\left(chemicaldesorption\right):2\;M-H^{*}\to H_{2}+M$$

In alkaline media (Figure 11b), the initial Volmer step requires overcoming water‐dissociation barrier (Equation (4)), which often becomes the rate‐limiting step.^[^ ^132^ ^]^ During hydrogen desorption, the hydrogen binding free energy (∆G_H*_) determines the preferred reaction pathway: lower ∆G_H*_ favours the Tafel mechanism (Equation (5)), whereas higher ∆G_H*_ promotes the Heyrovsky route (Equation (6)).

(4) $$Volmer\left(adsorption\right):H_{2}O+e^{-}+M\to M-H*+OH^{-}\left(RDS\right)$$

(5) $$Tafel\left(chemicaldesorption\right):2\;M-H*\to H_{2}+M$$

(6) $$\begin{array}{ccc} Heyrovsky\left(electrochemicaldesorption\right): & & M-H*+H_{2}O+e^{-}\\ & & \;\to H_{2}+M+OH^{-} \end{array}$$

Hydrogen adsorption strength is a core factor regulating HER, which exhibits the classical volcano‐type relationship with catalytic activity. Moderate hydrogen binding energies achieve a balance between proton activation in Volmer step and hydrogen desorption in Heyrovsky/Tafel steps. In neutral and alkaline media, hydrogen spillover mediated by support defects effectively mitigates intrinsic limitations such as insufficient proton supply and high water‐dissociation barriers. Nevertheless, hydrogen spillover itself encounters substantial kinetic and thermodynamic challenges (Figure 11c). The process comprises spillover and migration stages, requiring not only the overcoming of initial H‐transfer barriers but also smooth interfacial migration. To address this, some strategies such as heteroatom doping, alloying, phase optimization, and defect engineering have been developed to rationally adjust electronic structure and lower hydrogen spillover barriers for improving HER performance.

For example, Fu et al. designed a Ni_3_S_2_/Cr_2_S_3_ composite catalyst that exploited the hydrogen spillover mechanism to bridge the kinetic gap between water dissociation (Volmer step) and hydrogen recombination (Tafel step) in alkaline media, in which Cr_5c_ sites in Cr_2_S_3_ efficiently adsorbed and dissociated water molecules, while Ni_3_S_2_ promoted hydrogen recombination.^[^ ^133^ ^]^ Hydrogen spillover served as a bridge for rapid migration of H generated on Cr_2_S_3_ to Ni_3_S_2_ sites to prevent site poisoning at high densities. When used for HER, a current density of 3.5 A cm^−2^ and a overpotential of 251 mV were achieved in 1.0 m KOH (Figure 11d). Experimental and theoretical results further confirmed that the energy barrier along the hydrogen spillover pathway was significantly lower than the conventional Tafel route, and interfacial electronic coupling optimized the thermodynamic driving force for H migration. Yan et al. reported PtPd nanoclusters supported on CeO_2_ with short‐range hydrogen spillover to enhance HER performance in acidic media.^[^ ^103^ ^]^ Hydrogen migration from PtPd alloy surface to interface reduced reaction barrier, and the near‐zero hydrogen adsorption free energy at the interface further optimized kinetics. As shown in Figure 11e, the obtained catalyst achieved an overpotential of 5.7 mV with a long‐term stability. Both experiments and theoretical calculations indicated that hydrogen spillover could facilitate the migration of H* from strongly adsorbed metal sites (e.g., Pt) to neighbouring supports (e.g., metal oxides) rather than direct metal‐to‐support surface migration. In addition, Wei et al. anchored Pt nanoclusters on oxygen‐vacancy‐rich TiO_2_ (V*_O_*‐rich Pt/TiO_2_) for enhancing HER performance, and the mass activity reached 45.28 A mg^−1^ at ‐0.1 V (vs RHE), which were 16.7 and 58.8 times higher than V*_O_*‐deficient Pt/TiO_2_ and Pt/C, respectively (Figure 11f).^[^ ^95^ ^]^ Experimental and theoretical analyses revealed that oxygen vacancies reversed the direction of charge transfer (from TiO_2_ to Pt), electron‐enriching Pt and promoted hydrogen spillover from Pt to TiO_2_, thus optimizing *d*‐band center of Pt, lowering H adsorption energy, and enhancing HER performance. Sun et al. developed a photoelectrode combining cobalt‐doped LaFeO_3_ (Co‐LFO) with a 1T/2H MoS_2_ homojunction, in which the 2H phase formed a type‐II heterojunction with Co‐LFO to enhance photogenerated charge separation, while 1T‐MoS_2_ introduces active sites and hydrogen spillover pathways, improving proton transport and hydrogen adsorption/desorption kinetics.^[^ ^134^ ^]^ Therefore, the introduction of multiple regulatory mechanisms for manipulating hydrogen spillover is an effective solution to enhance HER properties.

Overall, the hydrogen spillover effect redefines the catalytic pathway and kinetics of HER by constructing efficient interfacial hydrogen transfer channels. Through the functional segregation and spatial cooperation of active sites, the highly active “donor” components preferentially catalyse the Volmer step for H* generation, while the adjacent “acceptor” supports or synergistic phases provide extended surfaces for H* migration and recombination. This dynamic interaction not only alleviates hydrogen‐induced site blocking and enhances turnover frequency, but also activates inert support surfaces. As a result, hydrogen spillover could accelerate reaction kinetics, reduce overpotential, and improve long‐term stability by preventing hydrogen‐induced deactivation.

### 7.2 OER

**Figure 12:**
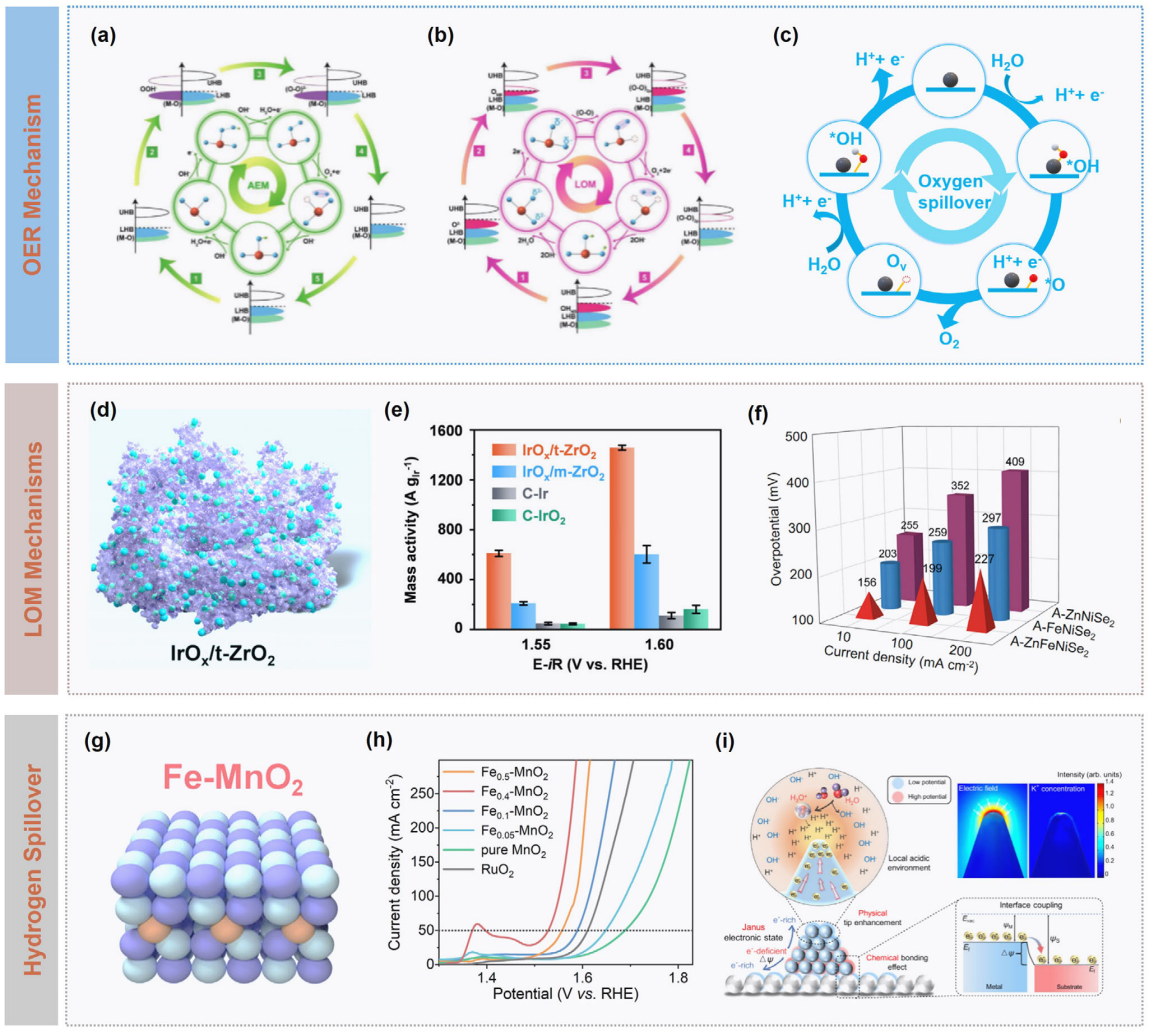
Spillover‐mediated mechanisms in OER. a,b) Schematic illustration of traditional non‐spillover catalyst reactions in (a) AEM and (b) LOM. Reproduced with permission.^[^ ^135^ ^]^ Copyright 2022, Wiley. c) oxygen spillover mechanism. d) Schematic illustration of oxygen spillover from active lattice oxygen on IrO_x_ nanoclusters to t‐ZrO_2_, and e) mass activity calculated. Reproduced with permission.^[^ ^126^ ^]^ Copyright 2025, Royal Society of Chemistry. f) Zn‐vacancy‐leached FeNiOOH enhances OH* spillover and lattice oxygen regeneration, with 156 mV overpotential at 10 mA cm^−2^. Reproduced with permission.^[^ ^53^ ^]^ Copyright 2025, Wiley. g) Schematic illustration of surface reconstruction of Fe doped MnO_2_ into α‐MnOOH, and h) LSV curves for OER. Reproduced with permission.^[^ ^137^ ^]^ Copyright 2025, Wiley. i) Schematic illustration of H* spillover‐enhanced HER/OER via Janus electronic states in Ir/NiPS_3_ (tip‐rich, interface‐poor electrons). Reproduced under a Creative Commons Attribution License 4.0 International License.^[^ ^138^ ^]^ Copyright 2024, The Authors.

In OER, the adsorbate evolution mechanism (AEM) and lattice oxygen‐mediated mechanism (LOM) are two core pathways. The conventional AEM pathway involves stepwise adsorption and transformation of reaction intermediates on active sites (**Figure** **12**a),^[^ ^135^ ^]^ as proceeded through consecutive proton‐electron transfer to generate O_2_. This mechanism does not directly consume lattice oxygen, thus maintaining structural stability of catalysts. In alkaline media, AEM follows a typical four‐electron transfer process; however, the multi‐step nature often results in sluggish kinetics and requires relatively high overpotentials. In contrast, the LOM pathway circumvents the limitations of traditional surface reactions (Figure 12b) by directly utilizing lattice oxygen from catalyst bulk for O_2_ formation, generating oxygen vacancies, which are subsequently replenished by hydroxide or water from the electrolyte. This pathway bypasses high‐energy‐barrier steps of AEM and thermodynamically exhibits superior reaction efficiency, enabling efficient catalysis at lower overpotentials. The key steps of LOM generally include:

(7) $$\begin{array}{ccc} Surfacehydroxylpre-adsorption: & & M*+OH^{-}\\ & & \;\to M-OH*+e^{-} \end{array}$$

(8) $$Latticeoxygenactivation:M-O_{L}\to M-O*+V_{O}$$

(9) $$O-\mathrm{Ocou}\mathrm{plin}\mathrm{gand}O_{2}\mathrm{rele}\mathrm{ase}:2\;M-O*\to 2\;M+O_{2}$$

Oxygen vacancy replenishment (by OH^−^ or H_2_O from the electrolyte):

(10) $$V_{O}+H_{2}O\to O_{L}+2H^{+}$$

(11) $$V_{O}+OH^{-}\to O_{L}+1/2H_{2}O$$

Although LOM significantly enhances OER activity, the formation of oxygen vacancies and the exposure of metal sites under high potentials can lead to over‐oxidation to soluble high‐valence states, causing structural collapse and performance decay. To address this challenge, the oxygen spillover effect facilitates the directional migration of active oxygen between donor and acceptor phases, offering an effective solution. As illustrated in Figure 12c, the cycle includes oxygen capture (acceptor vacancies capturing spillover oxygen), oxygen release (donor lattice oxygen preferentially participating in OER), and oxygen vacancy repair (water molecules replenishing vacancies). This mechanism activates inert sites, optimizes intermediate adsorption, prevents over‐oxidation of metal sites, and mitigates lattice oxygen consumption in the donor phase.

For example, Fang et al. constructed oxygen spillover on the tetragonal ZrO_2_ (t‐ZrO_2_) support to prepare IrO*_x_*/t‐ZrO_2_ catalysts (Figure 12d), which induced a mass activity of 1464.6 A g^−1^ at 1.6 V (Figure 12e) and a stable operation at 10 mA cm^−2^ for over 1000 h.^[^ ^126^ ^]^ It was confirmed that the support could efficiently dissociate water molecules and replenish IrO*_x_* lattice oxygen for preventing dissolution. Yin et al. leached Zn vacancies in FeNiOOH to promote hydroxyl spillover and lattice oxygen regeneration for stabilizing Fe sites and enhancing metal‐oxygen covalency, which presented an overpotential of 156 mV at 10 mA cm^−2^ and a stable operation above 200 mA cm^−2^ for over 500 h (Figure 12f).^[^ ^53^ ^]^

Notably, oxygen spillover and LOM exhibit a complex competitive and synergistic relationship. Oxygen spillover depends on active oxygen generated at metal sites, whereas LOM directly mobilizes lattice oxygen. Under strongly oxidative conditions, metal sites are prone to excessive oxygen coverage, suppressing spillover and shifting reaction toward LOM‐dominated pathways. Lin et al. used an Ir atomic lattice/MnO_2‐_ *_x_* model to demonstrate that Mn‐O*_x_* defects induced a locally oxygen‐rich surface layer for facilitating radical attacks and optimizing O─O coupling, and revealing the regulatory effect of surface oxygen coverage on reaction pathways.^[^ ^136^ ^]^

Furthermore, hydrogen spillover, while widely recognized for its role in HER, exhibits unique regulatory potential in OER. Hydrogen spillover enables the transfer of active hydrogen species between the catalyst surface and support, influencing surface reconstruction, electronic structure modulation, and intermediate adsorption, thereby enhancing overall reaction kinetics. For instance, Li et al. demonstrated that atomic‐level Fe doping modulated crystal reconstruction of ∆‐MnO_2_ to convert low‐activity γ‐MnOOH into highly active α‐MnOOH (Figure 12g).^[^ ^137^ ^]^ The resulting Fe─O─Mn bond distortion promotes electron migration and Mn^3+^ enrichment, enhancing oxygen spillover and significantly improving OER performance. Beyond doping strategies, electronic state regulation in metal‐support heterostructures has emerged as another effective route for promoting dual water‐splitting reactions. Liu et al. proposed and validated the Janus electronic state in an Ir/NiPS_3_ system, in which Ir nanoclusters exhibited electron‐rich states at apex regions and electron‐deficient states at interfaces (Figure 12i), forming a unique dual electronic distribution.^[^ ^138^ ^]^ This feature enables efficient HER and OER promotion under alkaline conditions through hydrogen‐spillover‐driven pathways, achieving 10 mA cm^−2^ of overall water‐splitting current density at 1.51 V in 1 M KOH with stable operation over 1000 h.

### 7.3 CO_2_RR

**Figure 13:**
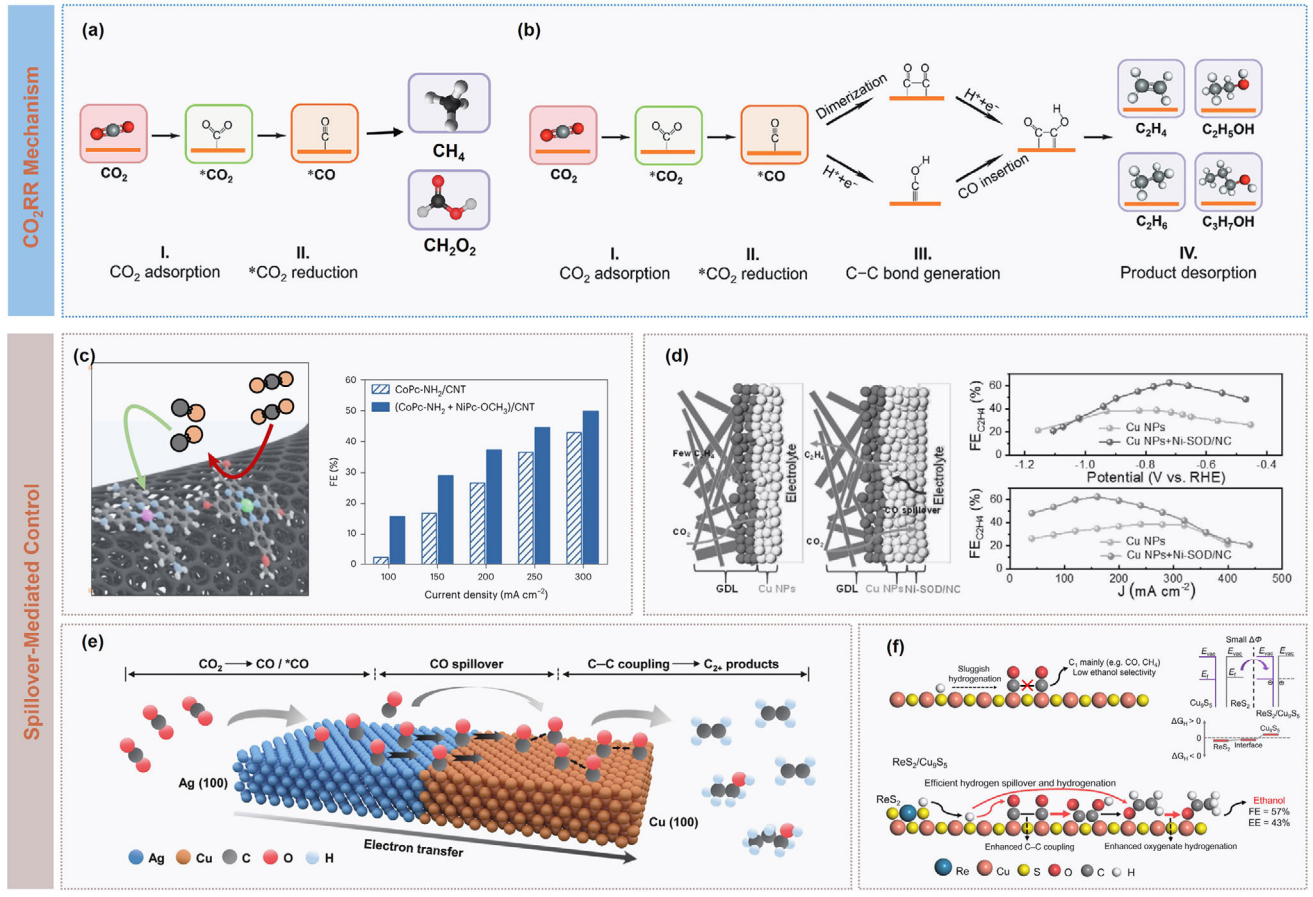
Spillover‐mediated mechanisms in CO_2_RR. a,b) Schematic illustration of main pathways to produce C_1_ products and C_2+_ products during CO_2_RR. Reproduced with permission.^[^ ^139^ ^]^ Copyright 2023, Wiley. c) Schematic illustration of CO spillover from NiPc‐OCH_3_ to neighboring CoPc‐NH_2_ for deep CO reduction to methanol, and FE at different densities. Reproduced with permission.^[^ ^61^ ^]^ Copyright 2025, Springer Nature. d) Schematic illustration of molecular‐scale CO spillover on Ni‐SOD/NC‐Cu enhancing CO coverage on Cu to promote C‐C coupling, and FE_C2H4_ at different potentials and densities. Reproduced with permission.^[^ ^141^ ^]^ Copyright 2023, Wiley. e) Schematic illustration of the CO_2_RR mechanism on Ag‐Cu Janus nanostructures. Reproduced with permission.^[^ ^143^ ^]^ Copyright 2022, Wiley. f) Schematic illustration of H* spillover‐enhanced ethanol selectivity on ReS_2_/Cu_9_S_5_ heterostructure. Reproduced with permission.^[^ ^144^ ^]^ Copyright 2025, Elsevier.

Electrochemical CO_2_RR represents an important catalytic conversion technology that is capable of transforming thermodynamically stable CO_2_ molecules into high‐value chemicals of varying carbon chain lengths. As depicted in **Figure** **13**a,b, CO_2_RR involves multi‐electron‐proton transfer processes that can yield C_1_ products such as CO, formate, and methane as well as C_2+_ products such as ethylene and ethanol.^[^ ^139^ ^]^ Mechanistically, the formation of C_1_ products typically requires 2–8 electron transfers, whereas the generation of C_2+_ products relies on more complex C─C coupling reactions. Due to the high stability of the C═O bond in CO_2_, activation often necessitates strongly negative potentials, and inevitably intensify the competitive hydrogen evolution reaction upon reduction. Hence, an in‐depth understanding on the interplay among active sites, interfacial properties, and intermediate adsorption is crucial for achieving efficient and selective CO_2_ conversion.^[^ ^140^ ^]^

For two‐electron reduction of CO_2_ to C_1_ products, the key step in methanol synthesis involves the further reduction of CO intermediates, identified as the RDS. This process is predominantly dictated by the equilibrium between CO adsorption and desorption on the catalyst surface, and increasing local CO concentration can significantly enhance protonation efficiency. For instance, Li et al. designed NiPc‐OCH_3_ sites to efficiently generate CO intermediates, and utilized a molecular‐scale CO spillover effect to transfer CO to neighbouring CoPc‐NH_2_ sites for deep reduction, resulting in a methanol Faradaic efficiency of 50% and a partial current density of 150 mA cm^−2^ (Figure 13c).^[^ ^61^ ^]^ It was observed that the increased coverage of CO intermediates confirmed the effectiveness of CO spillover mechanism. Moreover, adjusting reaction pressure enhanced methanol yield, reaching a peak Faradaic efficiency of 71%.

In contrast, the electrochemical production of multi‐carbon products involves more complex reaction pathways. Initially, CO_2_ is reduced at the catalyst surface through proton‐coupled electron transfer to form adsorbed CO. Subsequently, the neighbouring CO intermediates undergo surface C─C coupling to build multi‐carbon frameworks. This process possesses three kinetic limitations: i) the high activation barrier of CO_2_, ii) the limited CO coverage on copper surfaces, and iii) the sluggish C─C coupling kinetics. These factors collectively restrict selectivity and yield of C_2+_ products. To overcome this bottleneck, tandem catalysis strategies have been proposed, wherein metals such as Ag, Au, Pd, and Cu, efficiently generate CO intermediates at lower overpotentials, which then diffuse to the adjacent Cu sites for C─C coupling. It was known that Cu‐based catalysts exhibit negative CO adsorption energy and positive H adsorption energy, which can preferentially stabilize CO* intermediates and promote OCCO* formation, thermodynamically favouring multi‐carbon product generation. For example, Chen et al. developed a Ni‐SOD/NC‐Cu tandem catalyst, in which NiN_3_ sites were synergized with the neighbouring pyridinic nitrogen to generate CO efficiently at a low overpotential of −0.72 V, and molecular‐scale spillover considerably enhanced CO coverage on Cu surfaces for promoting C─C coupling, giving rise to an ethylene Faradaic efficiency of 62.5% and an industrially relevant current density of 160 mA cm^−2^ (Figure 13d).^[^ ^141^ ^]^

Further studies reveal that atomically dispersed Cu catalysts manifest good C_2+_ selectivity under high CO concentrations. For example, the Co‐Cu single atom catalyst delivered over 70% ethanol Faradaic efficiency at −0.8 V (vs RHE) with a stable operation for 18 h.^[^ ^142^ ^]^ Notably, the crystallographic orientation of the catalyst significantly affected product selectivity (Figure 13e). Specifically, owing to their distinct atomic arrangement, Cu (100) surfaces reduced the C─C coupling barrier compared with Cu (111), favouring C_2+_ formation.^[^ ^143^ ^]^ Liu et al. constructed a ReS_2_/Cu_9_S_5_ heterostructure to control H coverage, achieving an ethanol selectivity of 57% and an energy efficiency increase by 43% at −0.29 V).^[^ ^144^ ^]^ As illustrated in Figure 13f, the mechanism involves selective enhancement of *H adsorption at S sites on ReS_2_ surface without impeding *CO activation for facilitating the formation of *OCCOH intermediate.

Until now, significant advances have been made in understanding the mechanisms of C_1_ and C_2+_ product formation in CO_2_RR. Tailoring active site structure, electronic properties, crystal facets, and interfacial environments can effectively promote key intermediate generation and conversion, while suppressing competing reactions. Particularly, CO spillover, C─C coupling kinetics, and interfacial electronic structure optimization provide new strategies for achieving high‐efficiency, selective, and stable CO_2_RR.

In brief, spillover effects exhibit multiple advantages in CO_2_RR. First, by enabling the directional transfer of reaction intermediates across distinct active sites, the adsorption scaling limitations that are inherent to single‐site catalysts can be overcome. Second, the creation of spatially segregated cooperative catalytic architectures allows each active site to specialize in specific reaction steps, which can effectively improve the selectivity of C_2+_ product. Moreover, by facilitating timely removal of surface‐adsorbed species and modulating interfacial microenvironment, spillover processes maintain catalyst activity and structural integrity. Hence, these unique features establish spillover engineering as a important strategy for the rational design and optimization of highly efficient CO_2_RR electrocatalysts.

### 7.4 NRR

**Figure 14:**
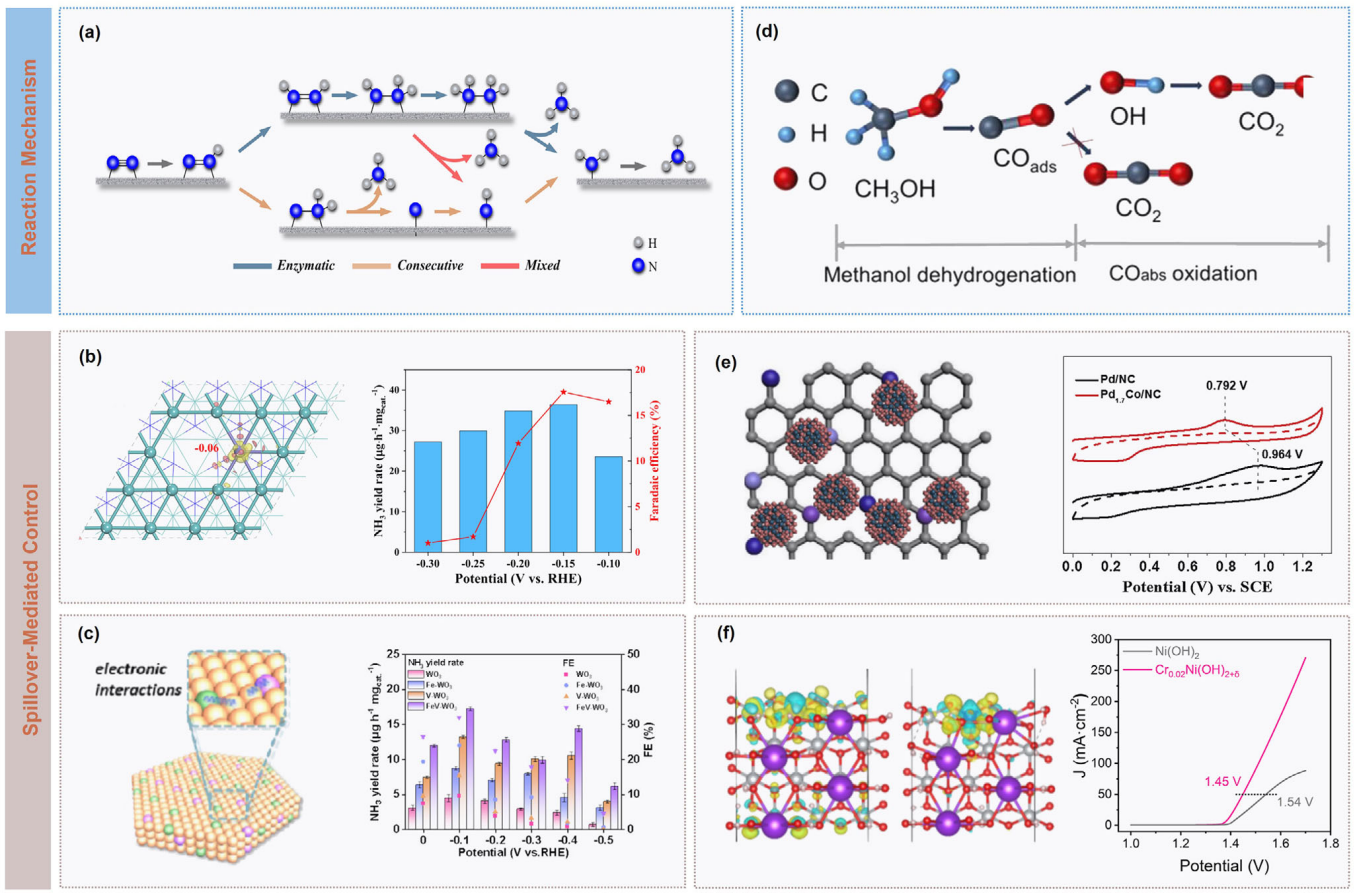
Spillover‐mediated mechanisms in NRR and MOR. a) Schematic illustration of NRR mechanisms. b) Schematic illustration of H* spillover from *OH to *N_2_ on Fe/SV‐Mo_2_N active center simultaneously adsorbing and activating OH^−^ and N_2_, and FE at different potentials. Reproduced with permission.^[^ ^147^ ^]^ Copyright 2023, Elsevier. c) Schematic illustration of the preparation of FeV‐WO_3_ catalyst, and ammonia yield rate and FE at different potentials. Reproduced with permission.^[^ ^148^ ^]^ Copyright 2025, Elsevier. d) Schematic illustration of the MOR mechanisms. Reproduced with permission.^[^ ^149^ ^]^ Copyright 2023, Elsevier. e) Schematic illustration of the bimetallic nanoalloys promoting hydrogen spillover, and the CO striping voltammograms of Pd_1.7_Co and Pd. Reproduced with permission.^[^ ^152^ ^]^ Copyright 2020, Elsevier. f) Charge density difference of the Cr_0.02_Ni (OH)_2+δ_ (100) surface, and LSV curves. Reproduced with permission.^[^ ^153^ ^]^ Copyright 2024, American Chemical Society.

Electrochemical NRR represents a sustainable route for ammonia synthesis and offers distinct advantages over nitrate/nitrite reduction processes.^[^ ^145^ ^]^ Although the latter benefits from the weaker N─O bond (≈204 kJ mol^−1^) compared to the N≡N triple bond (941 kJ mol^−1^) for faster reaction kinetics and higher Faradaic efficiency, its reliance on pre‐activated nitrogen sources severely limits practical application. In contrast, NRR directly utilizes atmospheric N_2_ as the feedstock, circumventing nitrogen sourcing and activation steps, demonstrating attractive green chemistry advantages.^[^ ^146^ ^]^ Nevertheless, the reaction mechanism involves six consecutive proton‐electron coupled transfer steps (**Figure** **14**a),^[^ ^147^ ^]^ in which the proton delivery from the electrolyte to adsorbed N_2_ molecules is a key bottleneck that constrains catalytic efficiency. Currently, there are still some challenges for NRR, such as sluggish kinetics, low conversion efficiency, limited catalyst stability, suboptimal ammonia selectivity, and competing HER.

To overcome these challenges, extensive research focuses on heterogeneous electrocatalysts with tailored electronic structures, including transition metals (e.g., Fe, Mo, Ru), noble metals (e.g., Au, Pd), metal oxides/nitrides (e.g., TiO_2_, VN), and their hybrid composites. These catalysts typically modulate *d*‐band structures, create electron‐deficient centers, or introduce interfacial synergistic effects to partially lower the activation barrier of N≡N and suppress HER. However, their practical application remains hindered by insufficient active site density and limited long‐term stability. Recently, catalyst design strategies based on spillover effects have demonstrated great potential as alternative solutions. By promoting N_2_ adsorption and selective protonation, these approaches effectively enhance proton transfer from the electrolyte to active sites. In alkaline media, hydrogen spillover occurs via adsorption‐activated OH^−^ species, allowing H atoms to migrate directionally from surface hydroxyls (OH) to activated N_2_, thereby accelerating nitrogen hydrogenation.

Sun et al. designed an Fe‐doped MoN single‐atom vacancy catalyst (Figure 14b) with the capability of simultaneously adsorbing hydroxyl and N_2_ species, in which Fe doping and vacancy introduction enhanced OH adsorption and weakened the O‐H bond, facilitating hydrogen migration to N_2_, while the resulting adsorbed O captured a proton to regenerate OH, establishing a sustainable hydrogen supply cycle.^[^ ^147^ ^]^ Using this approach, the free energy of the RDS for NRR was reduced to 0.22 eV, and the optimal catalyst exhibited an ammonia production rate of 36.4 µg h^−1^ mg^−1^ and a Faradaic efficiency of 17.6% under alkaline conditions (Figure 14b). Ji et al. introduced a low‐electronegativity bimetallic (Fe/V) co‐doping in WO_3_ catalyst (Figure 14c) to verify that Fe/V co‐doping could directionally tune electronic structure of high‐electronegativity W sites.^[^ ^148^ ^]^ This strategy increased electron filling in *d* orbitals, lowered N_2_ adsorption energy and enhanced activation efficiency. As shown in Figure 14c, at −0.1 V (vs RHE), the FeV‐WO_3_ catalyst achieved an ammonia production rate of 17.4 µg h^−1^ mg^−1^ and a Faradaic efficiency of 32.2%. In situ differential electrochemical mass spectrometry (DEMS) and electrochemical Raman spectroscopy studies confirmed the advantages of alternating reaction pathways and directly observed the dynamic evolution of key N_2_H_2_ intermediates, revealing possible migration of reactant species between Fe/V and W sites. Further theoretical calculations indicated that the Fe‐V synergy optimized the W *d*‐band center, lowered the free energy of RDS with a shift from N_2_ protonation to NH_2_NH_2_ decomposition. Besides, owing to electronic spillover altering competitive adsorption of H* and N_2_ on the catalyst surface, the designed bimetallic doping suppressed competing HER.

In summary, hydrogen spillover not only facilitates proton transfer and N_2_ activation in NRR but also provides a useful mean to regulate the RDS, offering both experimental and theoretical guidance for efficient ammonia synthesis under ambient conditions. While extensively studied in HER and CO_2_RR systems, its exploration in NRR remains limited, and the underlying mechanisms are not yet fully elucidated. Future research should consider multidimensional synergistic designs, such as interfacial polarization tuning, hierarchical porosity construction, or integration of in situ characterization with machine learning prediction. These approaches hold promise to advance electrochemical nitrogen reduction from laboratory‐scale studies toward scalable, green ammonia production.

### 7.5 MOR

In direct methanol fuel cells (DMFCs), anodic methanol oxidation reaction (MOR) constitutes a complex electrochemical process involving multiple electron transfers. As illustrated in Figure 14d, the widely accepted mechanism follows a dual‐pathway model, encompassing both indirect (CO pathway) and direct pathways.^[^ ^149^ ^]^ In the indirect pathway, methanol undergoes stepwise dehydrogenation on the catalyst surface to form strongly adsorbed CO intermediates, which block catalytic active sites and induce CO poisoning, and further oxidation to CO_2_ in the presence of hydroxyl species that are generated from water at high potentials. In contrast, the direct pathway bypasses strongly adsorbed CO, in which methanol is directly oxidized through soluble intermediates such as formaldehyde and formic acid. The competition between these two pathways influences overall catalytic activity and the resistance of catalyst poisoning. Fundamentally, MOR involves sequential dehydrogenation and oxidation through intermediates such as formaldehyde and formic acid, with the adsorption behaviour of CO intermediates serving as a key indicator of reaction efficiency. Although the thermodynamic potential of MOR is comparable to that of the HER, its multi‐step, six‐electron transfer nature induce kinetic limitations.^[^ ^150^ ^]^

The MOR pathway and rate are strongly influenced by electrolyte environment. In acidic media, CO intermediates, which are generated via multi‐step methanol dehydrogenation, strongly adsorb onto catalyst surfaces, causing catalyst poisoning and reduced reaction rates, and their complete oxidation to CO_2_ necessitates high overpotentials. Also, the deficiency of OH^−^ ions in acidic conditions requires water‐derived OH* to first adsorb on active sites, further lowering reaction efficiency. Conversely, alkaline media provide abundant OH^−^ ions, which could facilitate reaction kinetics and reduce catalyst poisoning, and carbonate species typically formed as final products. Wang et al. constructed a BiO*_x_* (OH)*_y_*‐Pt heterointerface via electrochemical reconstruction with the motivation to induce electron‐deficient Pt sites that weaken CO adsorption while enhance OH binding.^[^ ^151^ ^]^ Under alkaline conditions, the catalyst effectively removed toxic intermediates for achieving both high activity and stability.

Under strongly acidic or alkaline conditions, many catalysts are prone to dissolution, structural reconstruction, or redox transformations, resulting in unstable performance and limited long‐term operation. Pt‐ and Pd‐based materials remain the dominate MOR electrocatalysts. Nevertheless, they often require high onset potentials and are susceptible to CO poisoning. To address these challenges, further structural design on these catalysts is required. Hu et al. developed a low‐temperature reduction (UTR) strategy to synthesize ultrafine bimetallic nanoparticles, in which Co, Ni, Zn, and other metals were co‐reduced below their thermodynamic reduction temperatures, yielding Pd‐Co, Pd‐Ni, and Pd‐Zn alloys with an average particle size of ≈1.5 nm.^[^ ^152^ ^]^ The size effect and bimetallic synergy endowed these alloys with good MOR activity and CO tolerance. Notably, the Pd‐Co alloy achieved a peak current density of 2.8 mA cm^−2^ at −0.12 V, exhibited a specific activity approximately four times higher than that of pure Pd, and demonstrated a superior forward‐to‐backward current ratio. along with a superior forward‐to‐backward current ratio. Hydrogen temperature‐programmed reduction (H_2_‐TPR) measurements revealed that the Co reduction peak shifted from 230 to 210 °C in the presence of Pd, confirming low‐temperature reduction driven by hydrogen spillover. Also, X‐ray photoelectron spectroscopy (XPS) analysis indicated a negative shift of 0.4 eV in Pd 3d binding energy, evidencing electron transfer from Co to Pd and a pronounced alloying effect. Additionally, CO stripping tests showed 172 mV negative shift in CO oxidation potential compared with the pure Pd (Figure 14e), indicating significantly reduced CO adsorption strength.

Despite certain non‐noble metal catalysts demonstrating superior CO tolerance relative to Pt, their MOR activities are generally low, and reaction selectivity is poor, accompanied by commonly formed byproducts such as formic acid and formaldehyde. These limitations for non‐noble metal catalysts arise from unfavourable electronic structures and surface properties that hinder rapid conversion of key intermediates (CO, OH*) during multi‐electron transfer, leading to sluggish kinetics. Current research addresses these issues via some modulation strategies such as doping, alloying, and interface engineering to bridge the performance gap between non‐noble and noble metal catalysts. For instance, Qin et al. demonstrated that Cr doping in Ni‐based catalysts under alkaline conditions enhanced OH^−^ capture, and revealed that electron transfer from Ni to Cr lowered the electron density around Ni, strengthened OH^−^ adsorption and formed a locally OH^−^‐rich reaction interface.^[^ ^153^ ^]^ Also, Cr doping approach promoted water dissociation for generating a hydrophobic surface to reduce competitive adsorption of water molecules. This interfacial environment engineering enabled a current density of 50 mA cm^−2^ at 1.45 V (vs RHE), with nearly 100% formate selectivity (Figure 14f). Cui et al. employed a phosphotungstic acid adsorption‐decomposition strategy to prepare the WO_3_/C support, consisting of uniform dispersion of WO_3_ nanoparticles on carbon substrates, which enhanced the anti‐poisoning ability and catalytic activity of Pt catalysts.^[^ ^154^ ^]^ Hydrogen spillover from Pt surfaces to WO_3_ generated hydrogen tungsten bronze (H*_x_* WO_3_), freeing CO‐blocked active sites and accelerating methanol dehydrogenation via cyclic redox of H*_x_* WO_3_, leading to a MOR peak current of 254 mA mg^−1^.

Overall, MOR in DMFCs remains restricted by slow multi‐electron transfer, CO poisoning, and catalyst stability. While noble metal catalysts offer high activity, they are costly and prone to poisoning; non‐noble metal catalysts resist CO poisoning but suffer from low activity and selectivity. Through hydrogen spillover, interface engineering, doping, and alloying, electronic structures and reaction interfaces can be modulated to enhance active site utilization and reaction stability. Future efforts should focus on interfacial control, multifunctional synergy, and electronic structure design to develop highly active, CO‐tolerant, and cost‐effective MOR catalysts toward high‐efficiency and stable operation of DMFCs.

In summary, spillover effects are favourable for the MOR. Interfacial hydrogen transfer efficiently promotes C─H bond cleavage and accelerates dehydrogenation, while oxygen spillover from oxide supports facilitates the timely removal of surface‐adsorbed CO‐type poisons for enhancing anti‐poisoning capability. Furthermore, the construction of bifunctional catalytic interfaces enables spatial cooperation between methanol activation and intermediate oxidation, thus optimizing the overall reaction pathway. These mechanisms offer essential mechanistic guidance for the rational design of efficient and durable MOR catalysts.

## 8 Conclusion and Outlook

**Figure 15:**
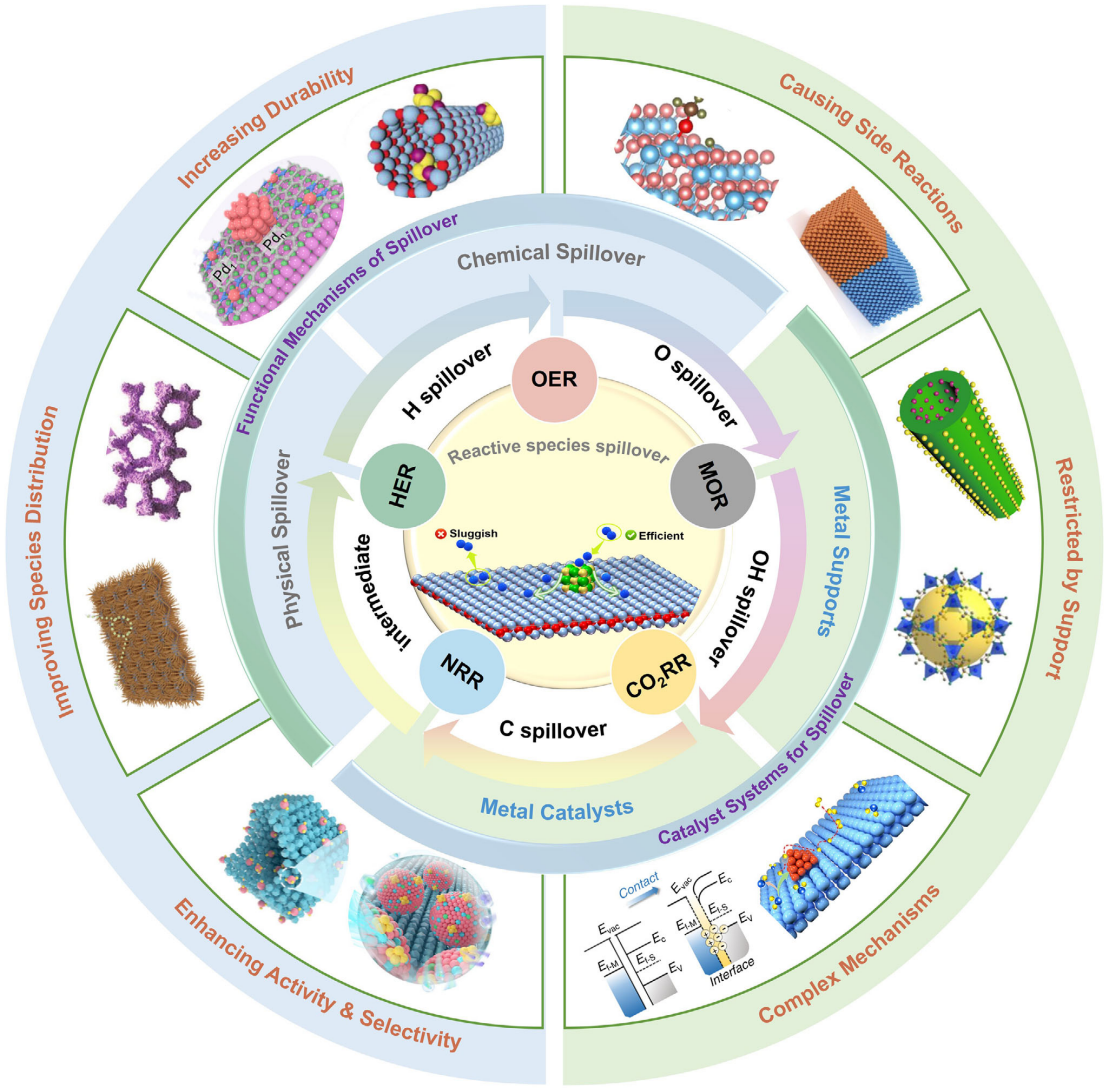
Schematic illustration of spillover effects for various electrocatalytic reactions.

Despite significant progress on spillover‐related research for electrocatalysis in recent years, a series of critical challenges remain, as depicted in **Figure** **15**, which spans from fundamental understanding to practical application. At the fundamental research level, the microscopic migration mechanisms of spillover species remain elusive, particularly the underlying structure‐activity relationships. At the process‐control level, achieving precise guidance of spillover migration pathways and targeted modulation of active sites necessitates overcoming the intrinsic limitations of conventional catalytic materials. Meanwhile, the dynamic structural evolution and stability degradation of electrocatalysts induced by spillover processes should be well addressed through multiscale materials design coupled with reaction engineering optimization.

In terms of characterization techniques, these existing techniques provide valuable insights into spillover phenomena, as summarized in **Table** **1**, and these approaches can be categorized into direct detection techniques and indirect analysis methods. Direct detection techniques, such as XPS, X‐ray absorption near‐edge structure (XANES), and angle‐resolved photoelectron spectroscopy (ARPES), can probe valence states and surface electronic structures of supports. However, such measurements typically yield averaged information and often require strict experimental conditions. In situ attenuated total reflection‐surface‐enhanced infrared absorption spectroscopy (ATR‐SEIRAS) is capable of capturing intermediate species under reaction conditions, yet it relies on specific substrates, and is highly susceptible to signal interference. STM offers atomic‐scale resolution, but it generally operates at cryogenic temperatures, which induces discrepancies from practical environments. Indirect analysis methods include temperature‐programmed desorption (TPD), temperature‐programmed reduction (TPR), UV–vis diffuse reflectance spectroscopy (UV–vis DRS), and various electrochemical techniques such as electrochemical impedance spectroscopy (EIS) and cyclic voltammetry (CV). TPD and TPR can infer the migration behavior of spillover species, but they struggle to preserve or directly observe spillover states, and their interpretations often suffer from ambiguity. UV–vis DRS can monitor valence state changes induced by spillover, but the signals typically originate from bulk‐phase variations and lack sufficient surface sensitivity. EIS and CV can provide insights into interfacial kinetics, however, they fall short of directly probing underlying chemical nature. On the theoretical side, DFT simulations can model spillover pathways and energy barriers at atomic scales, which can offer mechanistic support for experimental results. Nevertheless, the reliability of DFT results is strongly dependent on the choice of models and functionals, making it difficult to fully capture the complexity of practical reaction environments. Owing to the fact that identifying spillover effects under practical conditions is essential for accurately assessing catalytic performance and understanding reaction mechanisms, current research largely relies on indirect evidence. Therefore, developing techniques that offer atomic‐scale spatial resolution alongside real‐time monitoring represents a major challenge.

**Table 1:** Summary on characterization techniques for spillover‐related studies.

Technique	Judgment criteria	Literature example
Raman	Peak shift or intensity changes	Weakening W‐O peak in Ru‐WO_3‐_ *_x_* ^[^ ^81^ ^]^ Intensity change of Pd‐H peak^[^ ^41^ ^]^
H_2_‐T11811PR	Reduction peak shifts to lower temperature; Decrease in peak intensity	Ru lowers reduction temperature of MnO*_x_* ^[^ ^41^ ^]^ Accelerated CuO reduction in Pd‐Cu^[^ ^155^ ^]^
H_2_‐TPD	Appearance of new desorption peaks; Peak shift to lower temperature	H_2_ desorption peak shift in NiO/Cu^[^ ^156^ ^]^
XRD/STM/TEM	Lattice parameter change; Observation of surface atomic distribution	XRD peak shift of DOM‐H*_x_* WO_3_/Pt^[^ ^157^ ^]^ STM show H atom diffusion on MnO/Pt^[^ ^158^ ^]^
DFT Calculations	Hydrogen migration energy barrier; Hydrogen adsorption energy change	Reduced ΔG_H*_ for Pt/TiO_2_^[^ ^97^ ^]^ H* adsorption strength on Pt of P‐Pt_3_Co^[^ ^98^ ^]^
UV–vis DRS	Reflectance change; Absorption edge/extent change	Suppressed reduction state of Ni^[^ ^159^ ^]^
XANES	Change in metal valence state	3DOM WO_3_/Pt exhibits a broad absorption tail in the NIR region^[^ ^160^ ^]^
In situ ARPES	Electronic band structure change	Bandgap widening of graphene induced by C‐H bonds^[^ ^32^ ^]^
ATR‐SEIRAS	Change in peak intensity and position	Strongest *CO peak intensity on Cu NWs/NC@Ag PTFE, which almost disappears after ‐1.2 V^[^ ^64^ ^]^
XPS	Peak shift; Change in peak area	O 1s peak shift to higher binding energy, indicating aldehyde O as H binding site^[^ ^30^ ^]^
EIS	Change in hydrogen adsorption resistance (R*_i_*); Change in pseudo‐capacitance (C*_φ_*)	Largest Cφ for Pt_2_Ir_1_/CoP, enhanced adsorption; Lower Tafel slope derived from log R*_i_*, indicating accelerated H adsorption kinetics^[^ ^28^ ^]^
CV	Change in position and intensity of hydrogen adsorption/desorption peaks	Increased H desorption peak for Cu_1_/SiO_2_ with smaller slope of peak position vs scan rate^[^ ^161^ ^]^
pH/Temperature	Reaction order approximates 2.0; Activation energy derived from Arrhenius plot	Reaction order of 1.86 for Pt_1_@PW_12_@PC, activation energy of 20.9 kJ mol^−1[^ ^162^ ^]^

**Abbreviations**: TPR: Temperature‐Programmed Reduction; TPD: Temperature‐Programmed Desorption; DFT: Density Functional Theory; UV–vis DRS: UV–vis Diffuse Reflectance Spectroscopy; XANES: X‐ray Absorption Near Edge Structure; ARPES: Angle‐Resolved Photoemission Spectroscopy; ATR‐SEIRAS: Attenuated Total Reflection‐Surface Enhanced IR Absorption Spectroscopy; XPS: X‐ray Photoelectron Spectroscopy; EIS: Electrochemical Impedance Spectroscopy; CV: Cyclic Voltammetry.

In terms of electrocatalytic reactions, compared to these non‐spillover‐based systems (Tables S1 and S2, Supporting Information), current research on spillover effects has largely identified in prototypical systems such as HER and OER, as summarized in **Table** **2**, and their potential in other electrocatalytic processes (Table S3, Supporting Information), including CO_2_RR, NRR, and MOR, remains underexplored and requires systematic development. In these multiple proton‐electron transfer involved catalytic reactions with complex pathways, in which the concentrations of intermediates are low and their lifetimes are relatively short, active species are always rapidly consumed. As a result, spillover is difficult to be efficiently achieved and the direct observation remains challenging for these complex reactions. In addition, current catalysts suffer from intrinsic design limitations in constructing well‐matched carrier–active site interfaces, making it difficult to simultaneously balance high‐density active sites with efficient migration pathways. Depending on the advancement of state‐of‐the‐art characterization techniques that feature atomic‐scale spatial resolution and real‐time operando monitoring as well as the development of catalyst design strategies, spillover effects are expected to be more effectively exploited in a broader range of electrocatalytic reactions.

**Table 2:** Summary on electrocatalytic performance of spillover‐related catalysts.

Catalysts	Reaction	Electrolyte	Spillover	Overpotential [mV@j_10_]	Tafel slope [mV dec^−1^]	Stability [h / j]
Pd/CoNiP^[^ ^42^ ^]^	HER	1 M KOH	Hydrogen	19	21.2	100 / j_100_
Ir‐Nb/TiO_2_^[^ ^49^ ^]^	OER	0.5 M H_2_SO_4_	Oxygen	253	54.3	200 / j_10_
NiMo/WO*_x_* ^[^ ^72^ ^]^	HER	1 M KOH	Hydrogen	183@j_1000_	74	200 / j_1000_
ZnFeNiSe_2_^[^ ^53^ ^]^	OER	1 M KOH	Hydroxyl	156	33	500 / j_200_
PtPd/CeO_2_^[^ ^103^ ^]^	HER	0.5 M H_2_SO_4_	Hydrogen	5.7	15.6	400 / j_100_
Ru@Mn_3_O_4_^[^ ^107^ ^]^	HER	1 M KOH	Hydroxyl	17	17	40 / j_10_
Ru‐WO_3_@CF^[^ ^106^ ^]^	HER	0.5 M H_2_SO_4_	Hydrogen	17	27	200 / j_15_
Pt/BCN^[^ ^75^ ^]^	HER	0.5 M H_2_SO_4_	Hydrogen	26	24	60 / j_500_
RuO_2_/Nb_2_O_5_^[^ ^46^ ^]^	OER	0.5 M H_2_SO_4_	Oxygen	179	73	750 / j_10_
Fe*_x_*‐MnO_2_^[^ ^137^ ^]^	OER	1 M KOH	Oxygen	288@j_50_	39.6	1150 / j_100_
Pt‐NP/MoO*_x_* ^[^ ^76^ ^]^	HER	1 M KOH	Hydrogen	24	29.5	100 / j_10_
Dislocation‐rich Mo_2_C^[^ ^35^ ^]^	HER	1 M KOH	Hydrogen	61	32	150 / j_10_
Pt@WS_2_^[^ ^82^ ^]^	HER	0.5 M H_2_SO_4_	Hydrogen	31	27.16	100 / j_100_
IrO*_x_*/ZrO_2_^[^ ^126^ ^]^	OER	0.1 M HClO_4_	Oxygen	287	51.5	1000 / j_10_
RuNi/NC^[^ ^83^ ^]^	HER	1 M KOH	Hydrogen	12	30.85	1600 / j_10_
Ru‐Sn/SnO_2_ NS^[^ ^55^ ^]^	HER	1 M KOH	Hydroxyl	12	22.7	650 / j_10_
Pd@CoP/NF^[^ ^102^ ^]^	HER	1 M KOH+ Seawater	Hydrogen	249@j_1000_	37.02	500 / j_1000_
Ir‐Mn‐O*_v_* ^[^ ^136^ ^]^	OER	0.1 M HClO_4_	Oxygen	166	36	180 / j_100_
RuO_2_/MoO_3_^[^ ^47^ ^]^	OER	0.5 M H_2_SO_4_	Oxygen	167	65	300 / j_10_

In conclusion, spillover effects are significant for modulating electrocatalytic performance. By finely regulating the adsorption–desorption equilibrium of critical intermediates, reconstructing reaction pathways, expanding the distribution of active centers, and optimizing surface coverage of intermediates, spillover enables considerable improvement in reaction kinetics and selectivity. Through systematically reviewing the advances, it is crucial to partially elucidate the fundamental principles of active‐specie spillover and its mechanistic contributions to electrocatalysis. Future efforts should prioritize the in‐depth understanding on the spillover mechanisms, including the development of in situ/operando techniques capable of directly tracking the transient migration of spilled‐over species and quantitatively mapping their active sites to move beyond indirect evidence. It is expected that this timely review can provide theoretical guidance and practical insights for the rational design of highly efficient and selective catalysts for driving transformative progress in energy conversion fields.

## Conflict of Interest

The authors declare no conflict of interest.

## Supporting information



Supporting Information
