# Supplementary material for: Beyond Sabatier: Multiplicate Spillover Phenomena for Manipulating Catalytic Dynamics in Various Electrocatalysis
Source: Adv Sci (Weinh). 2026 Feb 3;13(13):e20177. doi: 10.1002/advs.202520177 (PMC12955903; doi:10.1002/advs.202520177)
Supplement: Supplementary file 1 — Supporting Information [file ADVS-13-e20177-s001.pdf]

Supporting Information

Beyond Sabatier: Multiplicate Spillover Phenomena for Manipulating Catalytic Dynamics in Various Electrocatalysis

Table S1. Electrocatalytic performance of non-spillover-based catalysts for OER and HER.

Catalysts	Reaction	Electrolyte	Overpotential ($mV@j_{10}$)	Tafel slope ($mV\ dec^{-1}$)	Stability (h / j)
**Ta-RuO_2[1]**	OER	0.5 M H_2SO_4	201	55	280 / j_{10}
**IrO_2@ATO[2]**	OER	0.5 M H_2SO_4	240	47.4	10 / j_{100}
**Ru/NdOC[3]l**	HER	1 M KOH	20	36	100 / j_{100}
**Pt4FeCoCuNi[4]**	HER	1 M KOH	20	31	50 / j_{10}
**CeO_2/Ce-Co_3O_4[5]**	OER	1 M KOH	216	87.6	100 / j_{10}
**(Mo, Co) P_x[6]**	OER	1 M KOH	213	25.37	480 / j_{100}
**W_1O/NiS[7]**	HER	1 M KOH	76	77	300/ j_{200}
**Pd_xS_y/1T-MoS_2[8]**	HER	0.5 M H_2SO_4	78	39.8	70/ j_{10}
**PdH@Ru[9]**	HER	1 M KOH	115 @ j_{50}	47.55	10/ j_{10}
**Fed-NiS_2/MXene[10]**	HER	1 M KOH	148	85	60/ j_{50}
**$Ni_{20}V$[11]**	HER	1 M KOH	88	54.3	200/ j_{10}
**RuO_2@TaO_x[12]**	OER	1 M KOH	163	62.6	100/ j_{10}
**Fe-Ni[13]**	OER	1 M KOH	186	31.4	1000/ j_{500}
**MoO_3-$Ni(OH)_2$[14]**	HER	1 M KOH	64	72.1	150/ j_{100}

Table S2. Electrocatalytic performance of non-spillover-based catalysts for CO_2RR, NRR, and MOR.

Catalysts	Electrolyte	Potentials (V)	Main products	FE %	Stability (h)
**ZnO-Ag** [15]	0.5 M $KHCO_3$	-0.93	CO	94	150
**fcc-2H-fcc Au-Cu JNS** [16]	0.1 M $KHCO_3$	-1.10	C_2H_4	56	10
**NiCu-SACs/N-C** [17]	0.5 M $KHCO_3$	-0.6	C_2H_5OH	92.2	/
**Au NBP−Cu JNCs** [18]	0.1 M $KHCO_3$	-0.98	C_2H_4	42	10
**Au−Cu** [19]	1 M KOH	-0.83	C_2H_5OH	40	7.5
**Cu/Au** [20]	0.1 M $KHCO_3$	-0.75	C_2H_5OH	60	90
**Pd/HsGDY** [21]	0.1 M KOH	-0.25	NH_3	44.5	/
**Au–Fe_3O_4 NPs** [22]	0.1 M KOH	-0.2	NH_3	10.54	2
**Pt_3Fe** [23]	1 M KOH	-0.05	NH_3	7.3	30
**FeV-WO_3** [24]	0.5 M K_2SO_4	−0.1	NH_3	32.2	24

Table S3. Electrocatalytic performance of spillover-based catalysts for CO_2RR, NRR, and MOR.

Catalysts	Electrolyte	Spillover species	Potentials (V)	Main products	FE %	Stability (h / j)
**CoCu** [25]	0.1 M $KHCO_3$	CO*	-0.8	C_2H_5OH	70	18
**ReS_2/Cu_9S_5** [26]	0.5 M $NaHCO_3$	H*	-0.29	C_2H_5OH	57.2±5.2	50
**NH_2-CdS/NMHCS** [27]	0.5 M $KHCO_3$	CO*	-0.9	CO	95	12
**Ni-SOD/NC** [28]	0.5 M $KHCO_3$	CO*	-0.72	C_2H_4	62.5	16
**CuCo-DSAC** [29]	0.5 M $KHCO_3$	COOH*	-0.4	CO	98.5	48
**Ag65-Cu_{35} JNS-100** [30]	0.1 M $KHCO_3$	CO*	-1.2	C_2H_4	54	10
**Pd-Cu** [31]	0.5 M $KHCO_3$	CO*	-1.0	C_2H_4	35	15
**Fe-Mo_2N** [32]	0.1 M KOH	H*	-0.22	NH_3	17.6	2
**Mo_1Fe_1Pd** [33]	KOH+KNO_3	N-containing intermediates	-0.7	NH_3	64.0	300

References

[1] X. Wang, Z. Li, H. Jang, C. Chen, S. Liu, L. Wang, M.G. Kim, J. Cho, Q. Qin, X. Liu, RuO_2 with Short-Range Ordered Tantalum Single Atoms for Enhanced Acidic Oxygen Evolution Reaction, *Adv. Energy Mater.* **2025**, *15,* 2403388.

[2] K. Zhou, Y. Wang, Z. Jiang, B. Dai, Z. J. Jiang, Ir/Mn Co-Mixing and Oxide-Support Interaction Modulation Through Plasma Promoted Asymmetric Oxygen Coupling for Stable Acidic Oxygen Evolution, *Adv. Mater.* **2025**, *37,* 2420159.

[3] T. Liu, Y. Chen, X. Wang, Y. Di, K. Müllen, Z. Zhang, F. Wang, Rare-Earth Oxychlorides as Promoters of Ruthenium Toward High-Performance Hydrogen Evolution Electrocatalysts for Alkaline Electrolyzers, *Adv. Mater.* **2025**, *37,* 2417621.

[4] Y. Wang, N. Gong, H. Liu, W. Ma, K. Hippalgaonkar, Z. Liu, Y. Huang, Ordering-Dependent Hydrogen Evolution and Oxygen Reduction Electrocatalysis of High-Entropy Intermetallic $Pt_4FeCoCuNi$, *Adv. Mater.* **2023**, *35,* 2302067.

[5] L.-X. Wang, C.-Z. Yuan, C.-H. Li, Y.-R. Zhang, F. Wu, L. Xin, Z. Wang, H. Yang, X. Shi, X. Zhang, K.N. Hui, S. Ye, Y. Chen, Interfacial Built-In Electric Field and Interatomic Charge Transfer Synergistically Boosting Oxygen Evolution on CeO_2/Ce-Co_3O_4 Electrocatalyst, *Adv. Funct. Mater.* **2025**, *35,* 2502122.

[6] Y. Che, J. Shang, Y. Zhang, S. Bo, J. Zhang, X. Qin, X. Liu, H. Sun, W. Zhou, Y. Jiang, X. Chen, S. He, D. Ma, F. Pan, Q. Liu, In Situ Engineering a Dual-Anion Rejection Interface for High-Efficiency Oxygen Evolution in Alkaline Seawater, *Adv. Energy Mater.* **2025**, *35,* 2507544.

[7] W.-G. Cui, X. Ren, S. Wang, Y. Zhang, Z. Li, K. Wang, F. Gao, Z. Shen, Y. Liu, X. Wang, Z. Wu, Y. Yang, D. Wang, H. Pan, Modulating the Structure of Interfacial Water via Oxygen-Coordinated Tungsten Single-Atom on Nickel Sulfide Slab to Boost Alkaline Hydrogen Evolution, *Adv. Energy Mater.* **2025**, *15,* e03257.

[8] H.D. Mai, S. Jeong, G.-N. Bae, N.M. Tran, J.-S. Youn, C.-M. Park, K.-J. Jeon, Pd Sulfidation-Induced 1T-Phase Tuning in Monolayer MoS_2 for Hydrogen Evolution Reaction, *Adv. Energy Mater.* **2023**, *13,* 2300183.

[9] X. Jiang, Y. Wang, J. Ding, C. Wang, Y. Tang, Y. Cao, W. Wang, G. Fu, Epitaxial Growth of PdH@Ru Hollow Nanobamboos for Efficient Hydrogen Evolution in Anion Exchange Membrane Electrolyzer, *Adv. Funct. Mater.* **2025**, *35,* 2414593.

[10] Y. Zhao, Y. Zhu, C. Xi, K. Hu, S. Han, J. Jiang, Manipulating NiS_2/MXene-induced Mott-Schottky Heterostructures via Precursor Doping Strategy for Promoting Alkaline Electrocatalytic Overall Water Splitting, *Appl. Catal. B Environ. Energy* **2025**, *378,* 125611.

[11] H. Jung, Y. Lee, K. Cho, T.Y. Kim, J. Song, H.S. Jung, S.I. Park, Y. Lee, J. Moon, W. Park, J. Nam, S. Park, W. Kim, J.W. Han, Strategic Design of Oxophilic Dopants for Active and Durable Alkaline Hydrogen Evolution Reaction Under Seawater, *Adv. Funct. Mater.* **2025**, e16387.

[12] J. Li, X. Yu, W.-H. Huang, Q. Zhang, K. Wei, X. Zhou, Y. Zhu, X. Zhong, M.-H. Yeh, N. Alonso-Vante, J. Ma, Multi-Step Screening-Guided Core-Shell RuO_2@TaO_x Nanorods Electrocatalyst for Acidic Oxygen Evolution Reaction, *Angew. Chem. Int. Ed.* **2025**, *64,* e202511750.

[13] L. Xue, B. Wang, J. Hu, C. Hou, C. Chen, Z. Zhu, X. Lv, J. Dang, Dynamic Self-Optimizing Reconstruction of Stainless Steel Fe-Ni Dual Sites Accelerates Catalytic Intermediate Coupling for High-Efficiency Oxygen Evolution Reaction at Industrial Current Density, *Adv. Funct. Mater.* **2025**, e16748.

[14] C. Ye, S. Yao, X. Xiao, P. Zhu, W. Zhang, X. Yang, C. An, Interfacial Electric Field-Mediated Stabilization of Unsaturated RuO_{2-x} Clusters on MoO_3-$Ni(OH)_2$ Heterostructure for Enhanced pH-Universal Hydrogen Evolution, *ACS Nano* **2025**, *19,* 26782-26790.

[15] Z. Zhang, G. Wen, D. Luo, B. Ren, Y. Zhu, R. Gao, H. Dou, G. Sun, M. Feng, Z. Bai, A. Yu, Z. Chen, "Two Ships in a Bottle" Design for Zn-Ag-O Catalyst Enabling Selective and Long-Lasting CO_2 Electroreduction, J. Am. Chem. Soc. **2021**, *143,* 6855-6864.

[16] Y. Ma, M. Sun, H. Xu, Q. Zhang, J. Lv, W. Guo, F. Hao, W. Cui, Y. Wang, J. Yin, H. Wen, P. Lu, G. Wang, J. Zhou, J. Yu, C. Ye, L. Gan, D. Zhang, S. Chu, L. Gu,

M. Shao, B. Huang, Z. Fan, Site-Selective Growth of fcc-2H-fcc Copper on Unconventional Phase Metal Nanomaterials for Highly Efficient Tandem CO_2 Electroreduction, *Adv. Mater.* **2024**, *36*, 2402979.

[17] S. A. Chala, K. Lakshmanan, W. H. Huang, A. W. Kahsay, C. Y. Chang, F. T. Angerasa, Y. F. Liao, J. F. Lee, H. Dai, M. C. Tsai, W. N. Su, B. J. Hwang, Cooperative dual single atom Ni/Cu catalyst for highly selective CO_2-to-ethanol reduction, *Appl. Catal. B Environ. Energy* **2024**, *358*, 124420,0926-3373.

[18] H. Jia, Y. Yang, T.H. Chow, H. Zhang, X. Liu, J. Wang, C.-y. Zhang, Symmetry-Broken Au–Cu Heterostructures and their Tandem Catalysis Process in Electrochemical CO_2 Reduction, *Adv. Funct. Mater.* **2021**, *31*, 2101255.

[19] T. Zhang, B. Zhang, Y. Zang, P. Zeng, Y. Li, H.J. Fan, A Selectivity Switch for CO_2 Electroreduction by Continuously Tuned Semi-coherent Interface, *Chem* **2024**, *10*, 2745-2760.

[20] S. Kuang, Y. Su, M. Li, H. Liu, H. Chuai, X. Chen, E.J.M. Hensen, T.J. Meyer, S. Zhang, X. Ma, Asymmetrical Electrohydrogenation of CO_2 to Ethanol with Copper-gold Heterojunctions, *Proc. Natl. Acad. Sci.* **2023**, *120*, e2214175120.

[21] Y. Guo, J. Liu, Q. Yang, P. Khemthong, Z. Huang, Y. Zhao, Z. Chen, B. Dong, X.-Z. Fu, J.-L. Luo, C. Zhi, Regulating Nitrogenous Adsorption and Desorption on Pd Clusters by the Acetylene Linkages of Hydrogen Substituted Graphdiyne for Efficient Electrocatalytic Ammonia Synthesis, *Nano Energy* **2021**, *86*, 106099.

[22] J. Zhang, Y. Ji, P. Wang, Q. Shao, Y. Li, X. Huang, Adsorbing and Activating N_2 on Heterogeneous Au–Fe_3O_4 Nanoparticles for N_2 Fixation, *Adv. Funct. Mater.* **2020**, *30*, 1906579.

[23] Y. Zhao, F. Li, W. Li, Y. Li, C. Liu, Z. Zhao, Y. Shan, Y. Ji, L. Sun, Identification of M-NH_2-NH_2 Intermediate and Rate Determining Step for Nitrogen Reduction with Bioinspired Sulfur-Bonded FeW Catalyst, *Angew. Chem. Int. Ed.* **2021**, *60*, 20331-20341.

[24] Z. Ji, X. Zhang, W. Gan, Y. Xue, X. Duan, Z. Zhou, Z. Han, S. Zhao, B. Feng, C. Li, M. Xu, G. He, Low Electronegativity Bimetallic Doping Modulates

Electrocatalytic Nitrogen Reduction Reaction Pathways for Synergistic Multi-objective Optimization, *Chem. Eng. J.* **2025**, *515*,163919,1385-8947.

[25] S.A. Chala, R. Liu, E.O. Oseghe, S.T. Clausing, C. Kampf, J. Bansmann, A.H. Clark, Y. Zhou, I. Lieberwirth, J. Biskupek, U. Kaiser, C. Streb, Selective Electroreduction of CO_2 to Ethanol via Cobalt–Copper Tandem Catalysts, *ACS Catal.* **2024**, *14*, 15553-15564.

[26] Z. Liu, L. Qin, A. Tang, H. Zhang, H. Cao, G. Zheng, ReS_2/Cu_9S_5 Interface Triggers Hydrogen Spillover for Boosting CO_2 Electroreduction to Ethanol with High Energy Efficiency, *Chem. Eng. J.* **2025**, *519*, 165341.

[27] R. Li, F. Xie, P. Kuang, T. Liu, J. Yu, Amino-Induced CO_2 Spillover to Boost the Electrochemical Reduction Activity of CdS for CO Production, *Small* **2024**, *20*, 2402867.

[28] J. Chen, D. Wang, X. Yang, W. Cui, X. Sang, Z. Zhao, L. Wang, Z. Li, B. Yang, L. Lei, J. Zheng, L. Dai, Y. Hou, Accelerated Transfer and Spillover of Carbon Monoxide through Tandem Catalysis for Kinetics-boosted Ethylene Electrosynthesis, *Angew. Chem. Int. Ed.* **2023**, *62*, e202215406.

[29] Y. Yang, W. Zhang, G. Wu, Q. Huang, J. Wen, D. Wang, M. Liu, Electronic Structure Tuning in Cu-Co Dual Single Atom Catalysts for Enhanced COOH* Spillover and Electrocalytic CO_2 Reduction Activity, *Angew. Chem. Int. Ed.* **2025**, *64*, e202504423.

[30] Z. Lyu, S. Zhu, L. Xu, Z. Chen, Y. Zhang, M. Xie, T. Li, S. Zhou, J. Liu, M. Chi, M. Shao, M. Mavrikakis, Y. Xia, Kinetically Controlled Synthesis of Pd-Cu Janus Nanocrystals with Enriched Surface Structures and Enhanced Catalytic Activities toward CO_2 Reduction, *J. Am. Chem. Soc.* **2021**, *143*, 149-162.

[31] Y. Ma, J. Yu, M. Sun, B. Chen, X. Zhou, C. Ye, Z. Guan, W. Guo, G. Wang, S. Lu, D. Xia, Y. Wang, Z. He, L. Zheng, Q. Yun, L. Wang, J. Zhou, P. Lu, J. Yin, Y. Zhao, Z. Luo, L. Zhai, L. Liao, Z. Zhu, R. Ye, Y. Chen, Y. Lu, S. Xi, B. Huang, C.-S. Lee, Z. Fan, Confined Growth of Silver–Copper Janus Nanostructures with {100} Facets for Highly Selective Tandem Electrocatalytic Carbon Dioxide Reduction,

Adv. Mater. **2022**, *34*, 2110607.

[32] C.N. Sun, Y.B. Qu, Z.L. Wang, Q. Jiang, Hydrogen Spillover in Alkaline Solutions for Effective Nitrogen Fixation, *Chem. Eng. J.* **2023**, *471,* 144589.

[33] W. Ye, Y. Yao, X. Wei, M. Xu, S. Zhao, W. Wang, G. Jia, F. Dai, P. Gao, X. Lu, X. Li, B. Xi, N. Wang, S. Xiong, Continuous Intermediates Spillover Boosts Electrochemical Nitrate Conversion to Ammonia over Dual Single-Atom Alloy, *Angew. Chem. Int. Ed.* **2025**, *64,* e202509303.
